# Pleiotropic brain function of whirlin identified by a novel mutation

**DOI:** 10.1016/j.isci.2024.110170

**Published:** 2024-06-04

**Authors:** Carlos Aguilar, Debbie Williams, Ramakrishna Kurapati, Rasneer S. Bains, Philomena Mburu, Andy Parker, Jackie Williams, Danilo Concas, Hilda Tateossian, Andrew R. Haynes, Gareth Banks, Pratik Vikhe, Ines Heise, Marie Hutchison, Gemma Atkins, Simon Gillard, Becky Starbuck, Simona Oliveri, Andrew Blake, Siddharth Sethi, Saumya Kumar, Tanaya Bardhan, Jing-Yi Jeng, Stuart L. Johnson, Lara F. Corns, Walter Marcotti, Michelle Simon, Sara Wells, Paul K. Potter, Heena V. Lad

**Affiliations:** 1 MRC Harwell Institute, Mammalian Genetics Unit, Harwell Campus, Didcot, Oxfordshire OX11 0RD, UK; 2 Mary Lyon Centre at MRC Harwell, Harwell Campus, Didcot, Oxfordshire OX11 0RD, UK; 3 School of Biosciences, University of Sheffield, Sheffield, South Yorkshire S10 2TN, UK; 4 Neuroscience Institute, University of Sheffield, Sheffield, South Yorkshire S10 2TN, UK

**Keywords:** Biological sciences, Neuroscience, Molecular neuroscience

## Abstract

Despite some evidence indicating diverse roles of whirlin in neurons, the functional corollary of whirlin gene function and behavior has not been investigated or broadly characterized. A single nucleotide variant was identified from our recessive ENU-mutagenesis screen at a donor-splice site in whirlin, a protein critical for proper sensorineural hearing function. The mutation (*head-bob*, *hb*) led to partial intron-retention causing a frameshift and introducing a premature termination codon. Mutant mice had a head-bobbing phenotype and significant hyperactivity across several phenotyping tests. Lack of complementation of *head-bob* with *whirler* mutant mice confirmed the *head-bob* mutation as functionally distinct with compound mutants having a mild-moderate hearing defect. Utilizing transgenics, we demonstrate rescue of the hyperactive phenotype and combined with the expression profiling data conclude whirlin plays an essential role in activity-related behaviors. These results highlight a pleiotropic role of whirlin within the brain and implicate alternative, central mediated pathways in its function.

## Highlights

• Hyperactivity associated with moderate hearing loss in ENU-induced whirlin mutant
• Overexpression of C-terminus whirlin in the brain rescues hyperactive behaviors
• Transcriptional profile of whirlin across tissues underscores pleiotropy
• Qualitative deficit in the temporal processing of the acoustic stimulus

## Graphical abstract

**Figure:**
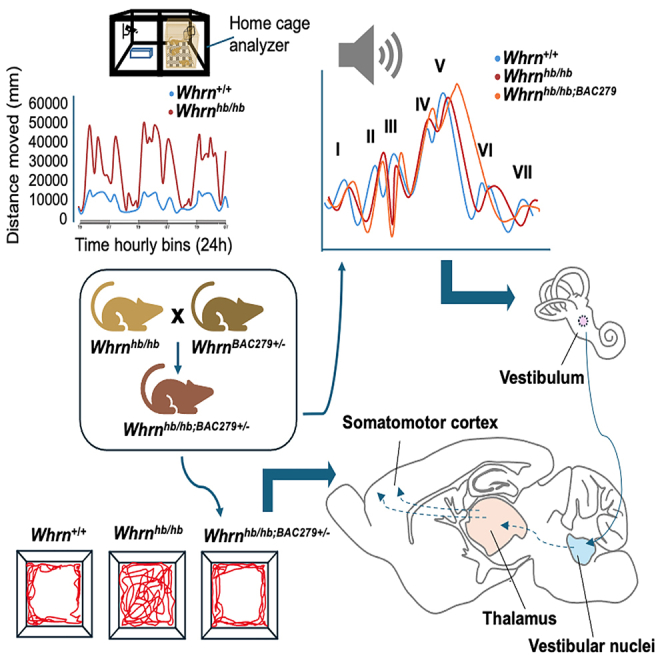

## Introduction

Whirlin is a cytoskeletal scaffold protein found to be essential for proper stereocilia elongation and development of hair cells within inner ears. Mutations in the gene are found in Usher syndrome type IIa leading cause of deafness and blindness in humans.^1^^,^^2^^,^^3^^,^^4^ The *whirler* mouse mutant carries a spontaneous deletion within the C-terminus of whirlin, resulting in profound deafness, severe head-tossing, and vestibular defects.^1^ Scanning electron microscopy (SEM) revealed that inner hair cell (IHC) stereocilia bundles were shorter and thicker in *whirler* mutants with their outer hair cell (OHC) stereocilia bundles abnormally rounded in shape. A BAC-transgene (BAC279), overexpressing wild-type protein of the C-terminal short isoform, rescued the head-tossing and circling phenotype in *whirler* mutants. Scanning electron microscope (SEM) analyses indicated the shortened IHC stereocilia defect found in mutants was repaired, but irregular OHC stereocilia bundle shape remained unchanged. Subsequent studies showed, using auditory brainstem response (ABR) and distortion product otoacoustic emissions (DPOE) that despite rescue of IHC stereocilia defects in the presence of BAC279, the profound deafness phenotype remains unaltered with an inherent deficit in OHC stereocilia function.^5^ These data strongly suggest that full-length whirlin is indispensable for sensorineural hearing function.

**Figure 1:**
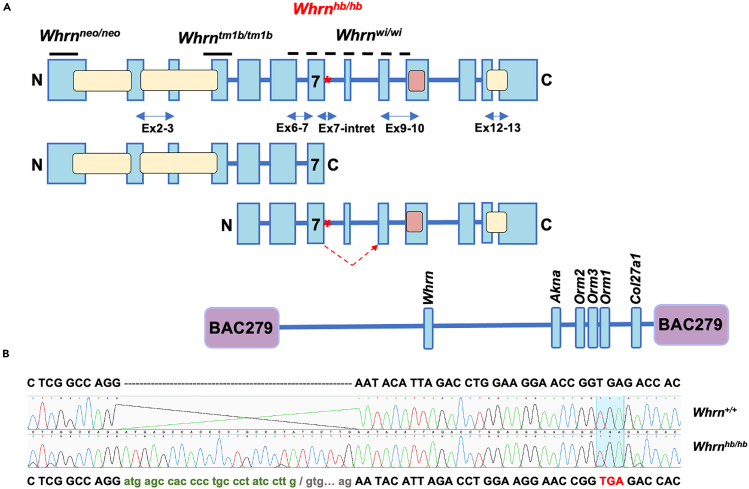
Main reported whirlin isoforms and mutations and partial intron retention found in *Whrn*^*hbhb*^ mutants (A) Canonical whirlin isoform (top) comprised of 13 exons showing the location of three PDZ-domains (yellow), a proline-rich domain (pink), and three reported whirlin mutations. *Whrn*^*hbhb*^ mutation is indicated (red asterisk) at the boundary of intron 7. Horizontal arrows (blue) below illustrate qPCR probes used for expression analyses. N-terminus (middle) and C-terminus (bottom) shorter isoforms are shown. Arrow (red) from exon 7 to exon 9 illustrated on the C-terminus isoform indicates skipping of exon 8, occurring in all tissue transcripts across each genotype. BAC279 transgene spans entire C-terminus short isoform and includes additional adjacent genes. (B) cDNA sequenced from *Whrn*^*+/+*^ and *Whrn*^*hb/hb*^ showing partial retention of intron 7 in the *Whrn*^*hb/hb*^ mutant transcript that introduces a shift in the reading frame. Readthrough into exon 9 (skipping exon 8 in both genotypes) and a frameshift in mutants results in a PTC highlighted (red) in sequence.

Multiple whirlin isoforms identified have partially complicated our understanding of the core functionality of numerous reported transcripts.^5^^,^^6^ The full-length isoform is around 900 amino acids long and contains three PDZ-domains and a proline-rich domain with alternative shorter isoforms considered to be functional at the C-terminus and N-terminus (Figure 1A) in mutants. Mouse knockout (KO) studies of the full-length isoform (*Whrn*^*neo/neo*^ and *Whrn*^*tm1b/tm1b*^) further demonstrated a functional role at the periciliary membrane complex (PMC) of photoreceptor cells and supported its role during early development within inner ears, at the tip-links and ankle-link complex of stereocilia.^2^^,^^4^^,^^5^^,^^6^^,^^7^ Mutants present with late onset retinal degeneration and moderate-to-severe high-frequency hearing loss. IHC stereocilia are unaffected in these mice, but OHC stereocilia bundle shape are irregular and paralleled those of the *whirler* mutant. From these studies, we can infer the full-length isoform of whirlin is critical for maintenance and development of OHC stereocilia bundle shape, while the C-terminus, short-isoform is required for IHC stereocilia elongation.

Varying domains across whirlin have been found to affect *in vitro* protein-protein interactions, due to mislocalization and destabilization of whirlin and its association with USH2 proteins within the Usher interactome.^8^^,^^9^^,^^10^^,^^11^^,^^12^^,^^13^ Whole mounts of the organ of Corti have shown that the C-terminus, short isoform, localizes to the tip-links at the site of mechanotransduction and ion-channel gating,^5^^,^^6^^,^^10^^,^^11^^,^^14^^,^^15^ whereas the full-length protein localizes to ankle- and interciliary-links of OHC stereocilia and its synaptic region.^3^^,^^6^^,^^7^

The impact both isoforms have on elongation of vestibular hair cell (VHC) stereocilia and their function has also been demonstrated in KO and *whirler* mutants.^1^^,^^5^^,^^6^^,^^16^^,^^17^ Distinctly shorter and wider VHC stereocilia and concomitant vestibular impairment was reflected by a reduced amplitude and increased latency of vestibular sensory evoked potential responses (VsEPs) in KO and *whirler* mutants,^6^^,^^17^ with vestibular defects more pronounced in the latter. Moreover, expression of C-terminal whirlin in the utricular macula and central crista ampularis was suggested to be sufficient to normalize vestibular defects associated with mutations in this region of the gene.^6^^,^^17^

The presence of a proline rich and three PDZ-domains strongly supports a functional role for whirlin at signaling junctions. Whirlin is known to be widely expressed in the brain^3^^,^^7^ and a study^18^ of CIP98, the rat homolog, found it localized within neurons specifically at axons and dendrites as well as around synapses. These findings suggested that formation of a complex between CIP98 and CASK may promote efficient vesicle trafficking at the synapse. Further reports suggest whirlin is a key stabilizing component of the axonal cytoskeleton and transport in myelinated axons,^19^ promoting effective compaction at paranodal junctions.^20^ DYSC, the Drosophila homolog of whirlin, is expressed presynaptically, often proximal to the active zone and found to be tightly linked to regulation of a calcium-activated potassium channel SLO.^21^^,^^22^ Investigations into the developmental plasticity of Na_v_-channels along the calyx of Held in rats,^23^ a major synapse located in the central auditory pathway, found a significant reduction in the clustering of these Na_v_-channels along the heminode when, additionally, they studied *whirler* mouse mutants, which was purported to result from improper sensory neuron formation in response to auditory stimuli. Despite some evidence supporting the diverse roles of whirlin in neurons, the functional corollary of whirlin gene function and behavior has not been deeply explored and remains to be fully characterized.

The utility of forward genetics approaches continues to be a powerful approach toward identifying and understanding the pleiotropic functions of genes. From a recessive ENU-mutagenesis screen,^24^ we identified a novel single nucleotide variant (SNV) at a donor splice site in whirlin. The mutation led to partial intron-retention at the splice junction causing a frameshift. Mutant mice had a head-bobbing phenotype, observed at weaning, and were significantly hyperactive—the mutant was therein named *head-bob* (*Whrn*^*hb/hb*^). The mutation resides within the deletion site of the *whirler* (*Whrn*^*wi/wi*^) mutation, which led us to investigate complementation of this novel ENU mutation and study the wider role of whirlin in behavior. Using broad-based phenotyping to characterize these mutants and transgenic crosses (Figure 1A), we found that *head-bob* (*Whrn*^*hb/hb*^) mutant mice had mild-moderate hearing loss that was much less severe than levels reported for *Whrn*^*wi/wi*^ and KO mice (*Whrn*^*neo/neo*^ and *Whrn*^*tm1b*/*tm1b*^). We discovered *head-bob* mutants displayed significant hyperactivity and that this behavioral phenotype was completely rescued by a transgene (BAC279) containing the C-terminal whirlin gene. Our findings implicate an alternative, essential function for C-terminal whirlin within neurons of the vestibulothalamic pathway. In addition, these data demonstrate the pleiotropic function of whirlin, shedding new light on its role in the brain and revealing novel behavioral mechanisms for this protein.

## Results

### Validating splice donor variant in whirlin

We identified a head-bobbing mutant from the Harwell recessive ENU mutagenesis screen and mapped the mutation to a 20 Mb region on chromosome 4. This mutation was thus named head-bob (*hb*). Whole-genome sequencing (WGS) identified an SNV at +1 of intron 7 in whirlin, introducing a splice donor site mutation. Sequencing mutant transcripts revealed the SNV resulted in a G>A base change and a 25bp retention of the intron in tissue extracts from inner ears, eyes, and brain. The frameshift led to a premature termination codon (PTC) within exon 9 (Figure 1B), implicating either nonsense mediated decay (NMD) of the transcript or production of a truncated protein. PCR over the mutation site to the C-terminus, revealed no alternative transcripts in mutant mice, suggesting NMD of the transcript. However, sequencing RT-PCR products over the mutation confirmed exon 8 (33bp length) was skipped in transcripts for all genotypes unless priming from this exon (data not shown) and spliced into exon 9. The intron retention sequence at the splice junction (UUG/GUG) featured a cryptic splice site.

### *Whrn*^*hb/hb*^ mutants display periodic bouts of hyperactivity in HCA and PIR

**Figure 2:**
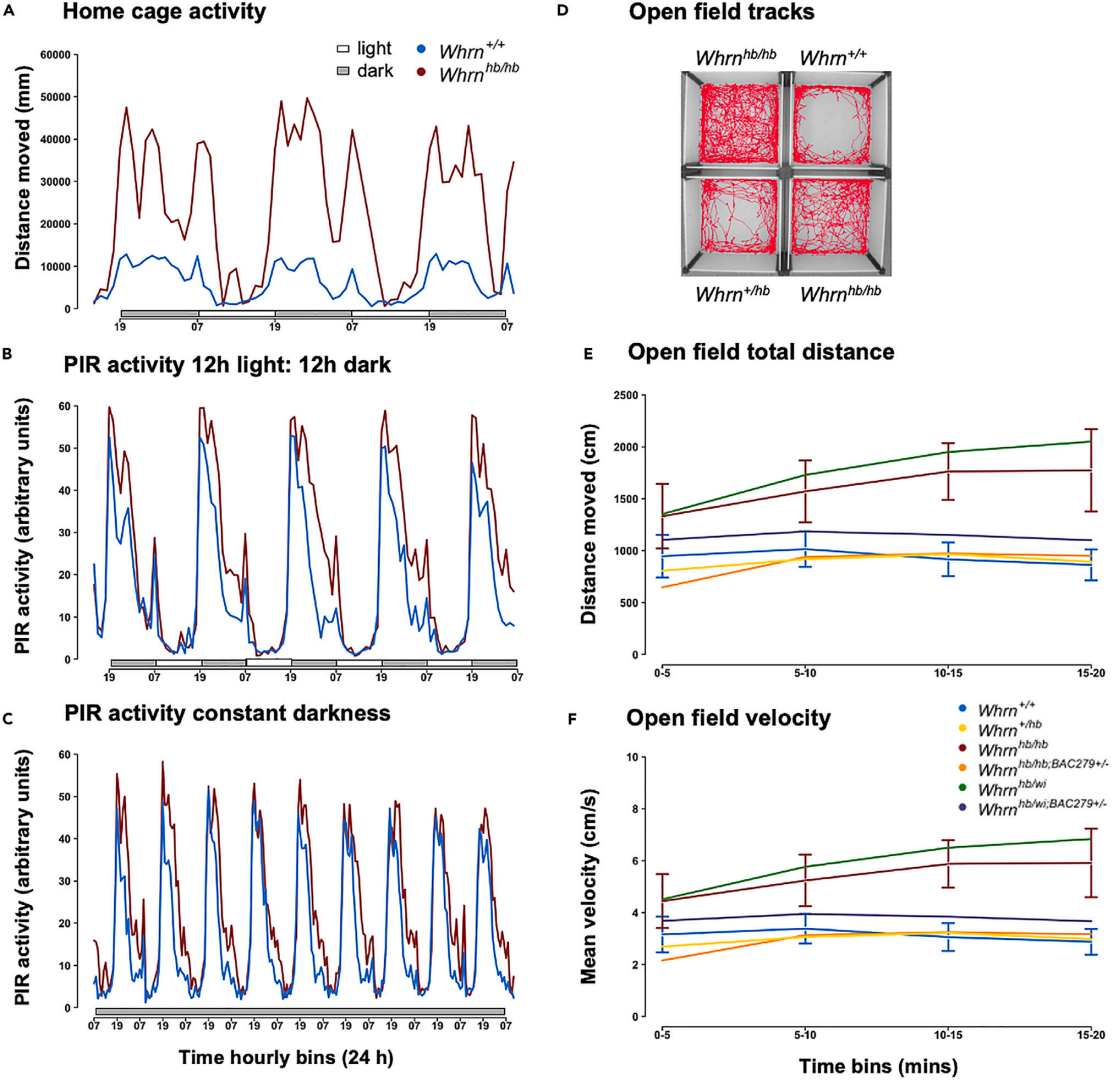
Activity measures from HCA, PIR, and open field (A) Data represent mean distance moved (mm) in the home cage for each hourly bin per genotype, plotted across 3 days. *Whrn*^*hb/hb*^ (*n* = 30) mutants displayed a significantly (*p* < 0.05) hyperactive phenotype during the dark and most active phase and on transitioning into the light phase compared with *Whrn*^*+/+*^ (*n* = 29). (B) Data are represented as mean PIR activity for each hourly bin per genotype plotted across 5 days in 12 h light:12 h dark in singly housed mice. Following an initial habituation phase, *Whrn*^*hb/hb*^ (*n* = 16) mutants showed similarly significant (*p* < 0.05) hyperactive profiles in PIR as in HCA compared with *Whrn*^*+/+*^ (*n* = 16) during the dark phase and on transitioning into the light phase. (C) Data represent mean PIR activity in constant darkness for each hourly bin per genotype plotted across 9 days. Profiles of hyperactivity paralleled HCA and PIR measures in 12 h light: 12 h dark at the start of the screen. Significant differences were attenuated toward the end of the screen. (D) Open field tracks illustrate lack of distinction between zone activity in *Whrn*^*hb/hb*^ (*n* = 22) mutants compared with *Whrn*^*+/+*^ (*n* = 20) and *Whrn*^*+/hb*^ (*n* = 14). (E) Data are represented as mean total distance moved (cm) during the open field test for each 5-min bin per genotype, +/− confidence interval width (95%) for *Whrn*^*+/+*^ and *Whrn*^*hb/hb*^. Activity measures confirmed *Whrn*^*hb/hb*^ and *Whrn*^*hb/wi*^ (*n* = 31) mutants were significantly hyperactive (*p* < 0.05) compared with *Whrn*^*+/+*^ and *Whrn*^*+/hb*^ in the final 3 bins. Transgenic lines, *Whrn*^*hb/hb;BAC279+/−*^ (*n* = 28) and *Whrn*^*hb/wi;BAC279+/−*^ (*n* = 12), recapitulate *Whrn*^*+/+*^ levels of activity and are significantly lower (*p* < 0.05) than activity of *Whrn*^*hb/hb*^ and *Whrn*^*hb/wi*^ mutants, validating a role for whirlin in activity. (F) Data represent mean velocity −/+ confidence interval width (95%) for *Whrn*^*+/+*^ and *Whrn*^*hb/hb*^, for each 5-min bin per genotype across the open field paralleled total distance results, reflecting *Whrn*^*hb/hb*^ hyperactivity indices and rescue in the transgenic mice.

We undertook continuous monitoring of *hb* mutant behavioral activity using the Home Cage Analyzer system (HCA; Actual Analytics, Edinburgh, UK) a passive monitoring platform to observe mouse behavior in their group housed, home environment. ANOVA for each hourly bin of activity in the home cage regressed against genotype and sex indicated genotype was mainly driving the activity phenotype (Table S1). In anticipation of lights off (at 1,900 h), activity increased significantly (*p* < 0.05, ANOVA) in both *Whrn*^*+/+*^ and *Whrn*^*hb/hb*^ (Figure 2A). Overall, *Whrn*^*hb/hb*^ were noticeably hyperactive during the dark phase and transitioning into the light phase. Periodic bouts of activity were significantly higher with three distinct episodes in *Whrn*^*hb/hb*^ mutants compared with *Whrn*^*+/+*^.

Movement with a passive infrared (PIR) field was used to determine activity across a subset of the cohort. Mice were housed individually within light- and sound-proof coffins. ANOVA for each hourly bin in the PIR regressed against genotype and sex revealed specific genotype and minimal sex effects (Tables S2 and S3). During the first 5 days, similar patterns of activity emerged as found in HCA when mice were under regular 12 h light:12 h dark conditions (Figure 2B). A phase of entrainment to single housing conditions can be initially seen in both *Whrn*^*+/+*^ and *Whrn*^*hb/hb*^ across the first 2 days when activity levels were highest. However thereafter, *Whrn*^*+/+*^ activity reduced, and *Whrn*^*hb/hb*^ continued to display increased episodes and duration of activity during the dark phase and on transitioning into the light phase. During constant darkness (Figure 2C), significant differences in activity between *Whrn*^*+/+*^ and *Whrn*^*hb/hb*^ were initially present in the active phase of their cycle. However, intriguingly these significant effects were attenuated toward the end of the screen, suggesting hyperactivity of mutants could be potentiated by light.

### Open field measures indicate hyperactivity across *Whrn*^*hb/hb*^ mutants

Open field behaviors are designed to capture the response of the mouse in a novel and aversive environment, determined by illumination across the central region of the open field. *Whrn*^*wi/wi*^ mice were omitted from open field testing due to the nature of the whirler phenotype and welfare concerns. Open field tracking data, total distance moved, mean velocity across the arena, and peripheral distance (Figures 2D, 2E, and S1C) indicated that *Whrn*^*hb/hb*^ mice and *Whrn*^*hb/wi*^ compound heterozygotes were significantly hyperactive compared to *Whrn*^*+/+*^, confirmed by multiple comparisons ANOVA (Table S4), paralleling outcomes from HCA and PIR. Crossing the transgene over the mutations (*Whrn*^*hb/hb;BAC279+/−*^ and *Whrn*^*hb/wi;BAC279+/−*^), to assess if expression of the short C-terminus isoform would affect the phenotype, resulted in diminished hyperactivity and activity levels comparable to *Whrn*^*+/+*^. The complete rescue of hyperactivity suggests that C-terminal whirlin plays a significant role in activity. Few differences were found for duration or distance moved in the center of the arena for the first three bins between *Whrn*^*+/+*^and *Whrn*^*hb/hb*^ mice but was significantly different between these genotypes in the final bin (Figures S1A and S1B), reflecting hyperactivity in the *Whrn*^*hb/hb*^ rather than specific anxiogenic behavioral effects.

### OCT revealed no deficits in the outer nuclear layer

The thickness of the outer nuclear layer (ONL), light-sensing part of the retina, across mutants, indicated no major effects between the reference strain (C3H*Pde6b*^*+*^) and mutant genotypes (Figures S2A and S2B). However, a significant reduction in ONL thickness was found for transgenic mice, which could be explained by genetic background variation in the *Whrn*^*hb/wi;BAC279+/−*^ mice or the presence of multiple copies of the wild-type fragment. These results will need further investigation on a genetically homogeneous background.

### Non-complementation and moderate deafness with temporal delay in ABR

**Figure 3:**
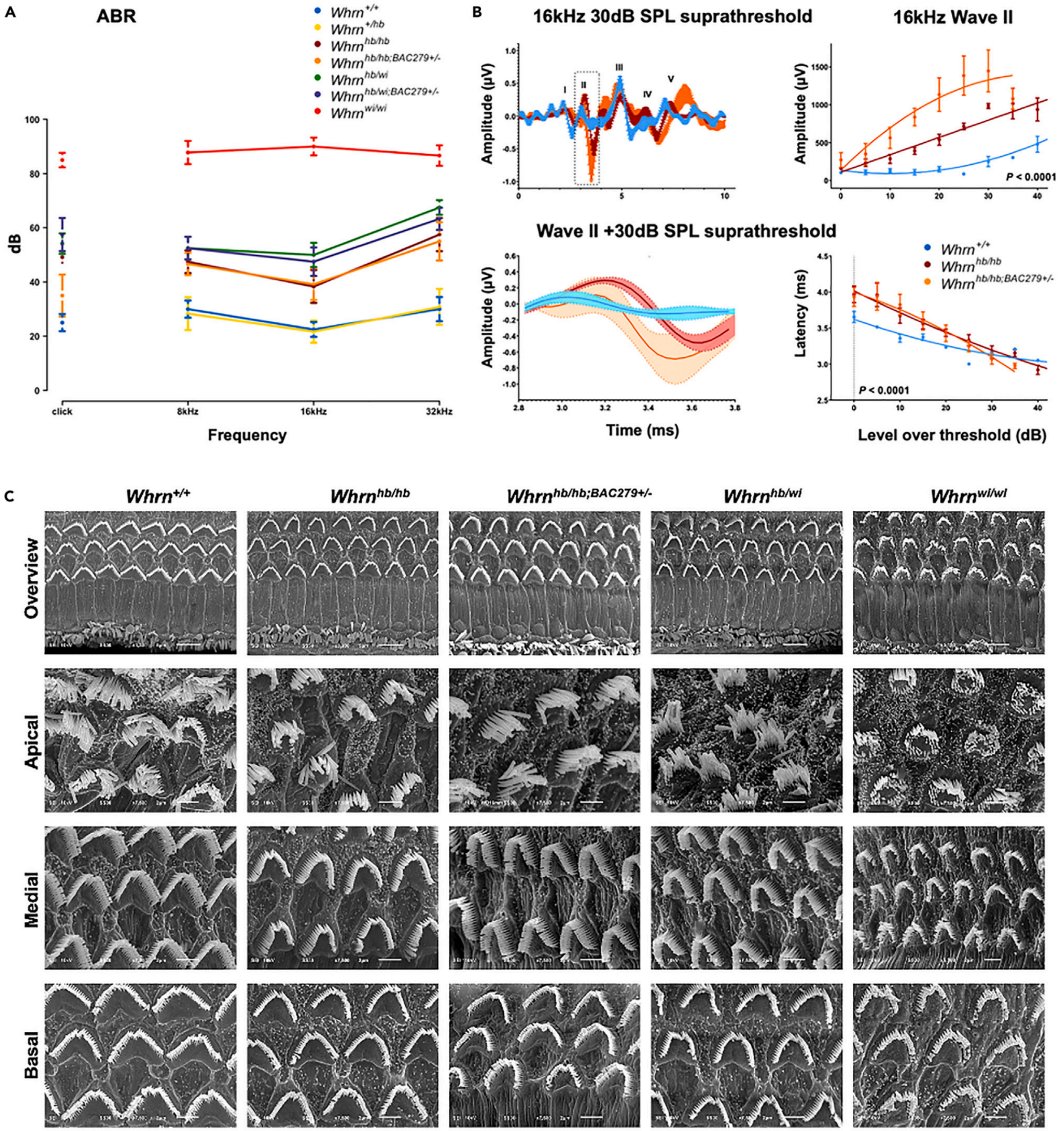
ABR, wave analysis and OHC stereocilia images taken using SEM (A) Auditory phenotyping using ABR across 7 genotypes and 3 different frequencies; data represent mean +/− confidence interval width (95%). Varying and significant (*p* < 0.01) levels of auditory hearing loss were observed across whirlin mutants with *Whrn*^*wi/wi*^ (*n* = 9) most profoundly deaf and *Whrn*^*hb/hb*^ (*n* = 6) and *Whrn*^*hb/wi*^ (*n* = 6) showing mild-moderate levels of hearing. Auditory loss was not rescued in the presence of the transgene, *Whrn*^*hb/hb;BAC279+/−*^ (*n* = 6) and *Whrn*^*hb/wi;BAC279+/−*^ (*n* = 6). *Whrn*^*+/+*^ (*n* = 6) and *Whrn*^*+/hb*^ (*n* = 6) were comparable. (B) Detailed analyses of amplitude and latency of the 16 kHz 30dB SPL suprathreshold trace (center) and along the stimulus series, corrected for threshold (right). At wave II (box on main waveform plot), the amplitude of the responses of *Whrn*^*hb/hb*^ and *Whrn*^*hb/hb;BAC279+/*^ were significantly different (*p* < 0.01) compared with *Whrn*^*+/+*^, and latency measures reflect a significant delay. Data are represented as mean ± SEM. (C) Representative SEM images of inner ears dissected at 1-month old. Overview images indicate few IHC stereocilia differences across genotypes except for *Whrn*^*wi/wi*^ where IHC stereocilia are short. OHC stereocilia bundles across mutants appear more rounded in shape and spaced apart compared with *Whrn*^*+/+*^. Few differences are observed across head-bob mutants; *Whrn*^*wi/wi*^ OHC stereocilia are sparse and shorter. Medial and basal layers illustrate pronounced shape and spatial differences of OHC stereocilia.

ABR was used to determine levels of hearing function and complementation of the combined whirlin mutations (Figure 3A). Click-stimulus showed some broad and significant differences (Table S5) in auditory response between mutant genotypes (*Whrn*^*hb/hb*^, *Whrn*^*hb/wi*^, *Whrn*^*hb/hb;BAC279+/−*^, *Whrn*^*hb/wi;BAC279+/−*^, and *Whrn*^*wi/wi*^), against wild types and heterozygotes (*Whrn*^*+/+*^and *Whrn*^*+/hb*^). However, frequency-specific tone-burst stimuli revealed some distinct phenotypic effects. *Whrn*^*+/+*^ and *Whrn*^*+/hb*^ responses demonstrated no hearing deficits, while *Whrn*^*wi/wi*^ mutants were profoundly deaf as expected. Strikingly, both *Whrn*^*hb/hb*^ and *Whrn*^*hb/wi*^ had mild-moderate hearing loss that confirmed non-complementation of the SNV. Both mutants carrying the transgene, *Whrn*^*hb/hb;BAC279+/−*^ and *Whrn*^*hb/wi;BAC279+/−*^, also had similar mild-moderate hearing loss, which was not rescued with overexpression of the transgene.

Visualization of ABR waveforms across a subset of genotypes at 30 dB (SPL) suprathreshold of the 16 kHz response, suggested an altered wave II in *Whrn*^*hb/hb*^ and *Whrn*^*hb/hb;BAC279+/−*^ mice (Figure 3B). Detailed analyses confirmed there was a significantly increased amplitude (nV) of wave II in both *Whrn*^*hb/hb*^ and *Whrn*^*hb/hb;BAC279+/−*^ and a marked temporal delay in the response latency at lower intensities. This suggests that *Whrn*^*hb/hb*^ mice have an inherent deficit in the precision of the relay of the encoded auditory stimulus beyond the cochlea.

### SEM comparable across mutants carrying the *hb* allele

Organ of Corti harvested from 6 genotypes (Figure 3C) at one month old, were prepared and imaged using SEM. The IHC stereocilia of *Whrn*^*+/+*^ and *Whrn*^*hb/hb;BAC279+/−*^ appeared similar while subtle differences were observed between remaining mutants carrying the *hb* allele, in which IHC stereocilia were marginally shorter and thicker. However, IHC stereocilia of *Whrn*^*wiwi*^ were significantly shorter and thicker than wild type as previously reported.^1^^,^^5^ OHC stereocilia were notably affected within the medial and basal turns of the organ of Corti across all mutants, where bundles appear more rounded and space between bundles set apart compared to wild type, with the outermost row of OHC stereocilia somewhat overextended. *Whrn*^*wiwi*^ OHC stereocilia were the most severely affected being shorter and having fewer complete rows of hair cell stereocilia present.

### Potential loss of Ca^2+^ sensitivity of the MET current in whirlin mutants

Mechanoelectrical transducer (MET) currents were recorded from OHCs by displacing stereocilia bundles in excitatory and inhibitory directions using piezo-driven fluid jets.^25^^,^^26^ Excitatory displacement (toward tallest stereocilia) from hyperpolarized membrane potentials in OHCs from P5-P7 mice elicited a large inward MET current (Figures S3A–S3C). Maximal MET currents of OHCs, in 1.3 mM Ca^2+^, for *Whrn*^*+/+*^ (−1370 ± 148 pA at −121 mV; *n* = 4) was not significantly different (*p* = 0.6965, one-way ANOVA) compared with *Whrn*^*+/hb*^ (−1292 ± 73 pA; *n* = 13) and *Whrn*^*hb/hb*^ (−1180 ± 232 pA; *n* = 5) mice (Figure S3D). Comparable maximal MET currents were found at −121 mV in P5-P8 OHCs, across whirlin mutant genotypes (*Whrn*^*hb/wi*^: −842 ± 139 pA, *n* = 6; *Whrn*^*hb/hb*^: −785 ± 181 pA, *n* = 6; *Whrn*^*wi/wi*^: −690 ± 103 pA *n* = 10, *p* = 0.7083, one-way ANOVA, Figures S3E–S3H). The resting open probability (*P*_*open*_) of MET channels at −121 mV was similar at P5-P7 between wild type, heterozygotes, and homozygotes (*Whrn*^*+/+*^: 8.3 ± 0.7%, *n* = 4; *Whrn*^*+/hb*^: 6.8 ± 0.3%, *n* = 13; *Whrn*^*hb/hb*^: 8.1 ± 0.4% *n* = 5, *p* = 0.056). In P8 OHCs, MET current *P*_*open*_ was also comparable across whirlin mutants at −121 mV (*Whrn*^*hb/wi*^: 9.2 ± 1%, *n* = 6; *Whrn*^*hb/hb*^: 7.5 ± 1% *n* = 6; *Whrn*^*wi/wi*^: 6.3 ± 1%, *n* = 10, *p* = 0.2929). At positive membrane potentials (+99 mV), MET currents exhibit a larger resting transducer channel *P*_*open*_, resulting from a decreased driving force for Ca^2+^ influx and reducing adaptation.^26^^,^^27^^,^^28^ *P*_*open*_ was significantly larger in *Whrn*^*+/+*^ (58 ± 5%, *n* = 4) at +99 mV compared with *Whrn*^*+/hb*^ (39 ± 3%, *n* = 13, *p* < 0.05) and *Whrn*^*hb/hb*^ (36 ± 4% *n* = 5, *p* < 0.05; one-way ANOVA, Tukey post-hoc). Interestingly, at +99 mV in P8 OHCs, *P*_*open*_ across all mutants is significantly reduced across all mutants (*Whrn*^*hb/wi*^: 40.2 ± 4%, *n* = 6; *Whrn*^*hb/hb*^: 32.5 ± 6% *n* = 6; *Whrn*^*wi/wi*^: 22.9 ± 5%, *n* = 10, *p* = 0.0645) compared to responses in P7 OHCs of *Whrn*^*+/+*^. These outcomes further validate non-complementation of the *hb* allele and highlight potential loss in the Ca^2+^ sensitivity of the MET channel across whirlin mutations.

### Mutant IHCs lack maturation of electrophysiological traits

The onset of adult-like characteristics in OHCs of mouse cochlea occurs at ∼ P8 with expression of negatively activated K^+^ current *I*_K,n_,^29^ which drives the largest K^+^ current in OHCs through KCNQ4 channels.^30^ K^+^ currents from apical coil OHCs were elicited by applying a series of depolarizing voltage steps in 10 mV increments from −124 mV, starting at a holding potential of −84 mV. We found adult OHCs of Whrn^+/+^ and mutant mice (*Whrn*^*hb/hb*^, *Whrn*^*hb/wi*^, and *Whrn*^*wi/wi*^) exhibit comparable current profiles (Figures S4A–S4F). The magnitude of isolated *I*_K,n_ was not significantly different in OHCs between the *Whrn*^*+/+*^, *Whrn*^*+/hb*^, and *Whrn*^*hb/hb*^ mice (*p* = 0.0579: Figure S4G, one-way ANOVA).

IHCs begin to acquire their adult-like basolateral membrane characteristics at ∼ P12,^31^ which include the expression of *I*_K,n_,^32^^,^^33^ and fast activating large conductance Ca^2+^ activated K^+^ current (*I*_K,f_).^32^^,^^34^ Potassium currents in mature IHCs (P30-P62) were elicited by applying depolarizing voltage steps in 10 mV nominal increments from −144 mV, starting at a holding potential of −64 mV (Figures S5A–S5D). We found that *I*_K,f_ was significantly reduced in whirlin mutants (*I*_K,f*,*:_ *p* < 0.0001, one-way ANOVA, Figure S5E). A significant difference in *I*_K,n_ was only detected between *Whrn*^*+/+*^ and *Whrn*^*wi/wi*^ (*p* < 0.05). These results indicate OHCs appear to develop normally but, IHCs fail to acquire their mature-like basolateral membrane characteristics.

We additionally investigated whether adult IHCs synaptic activity was altered between *Whrn*^*+/+*^ and *Whrn*^*hb/hb*^ mice. Measures were estimated, using exocytosis as a proxy, to infer increases in IHC membrane capacitance (*ΔC*_*m*_) following a 50 ms depolarizing voltage.^31^^,^^35^^,^^36^ We found no significant differences between the IHCs of *Whrn*^*+/+*^ and *Whrn*^*hb/hb*^ mice (P25) for either the maximal size of the Ca^2+^ current (*Whrn*^*+/+*^: −96 ± 16 pA, *n* = 5; *Whrn*^*hb/hb*^: −104 ± 13 pA, *n* = 4) or, the corresponding *ΔC*_*m*_ (*Whrn*^*+/+*^: 12 ± 1 fF, *Whrn*^*hb/hb*^: 14 ± 2 fF; and, *I*_*Ca*_: *p* = 0.7027; *ΔC*_*m*_: *p* = 0.2332) (Figures S6A and S6B). Furthermore, synaptic transfer functions, correlating the peak *I*_*Ca*_ and *ΔC*_*m*_ at different membrane potentials,^36^^,^^37^ were similar between IHCs of *Whrn*^*+/+*^ and *Whrn*^*hb/hb*^ mice (Figure S6C).

### Expression profiling reveals pleiotropic transcription

**Figure 4:**
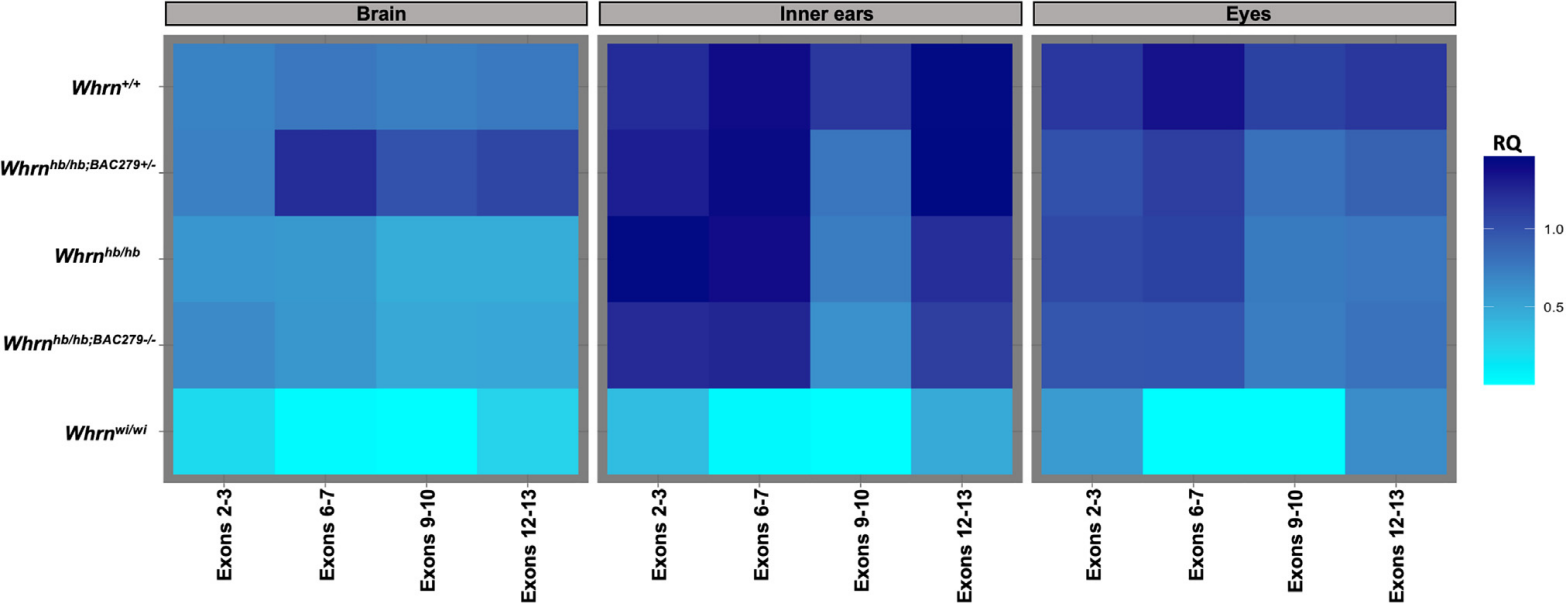
Relative quantification (RQ) of whirlin expression across the brain, inner ears, and eyes at P5-6 Data represent transformed relative quantities per probe across tissues. *Whrn*^*+/+*^ (*n* = 8) expression is marked within inner ears and eyes in contrast with brain tissue. Downstream of the mutation site, beyond intron 7, expression in *Whrn*^*hb/hb*^ (*n* = 8) and *Whrn*^*hb/hb;BAC279−/-*^ (*n* = 6) is significantly reduced (*p* < 0.05) comparative to expression levels of *Whrn*^*+/+*^ across all tissues yet appears to increase across exons 12–13 uniquely within the inner ears. An altered profile of expression is found only in brain transcripts from the *Whrn*^*hb/hb;BAC279+/−*^ (*n* = 11), overexpressing the C-terminal isoform, spanning the mutation site. Minimal impact of the transgene is seen across inner ears and eyes. *Whrn*^*wi/wi*^ (*n* = 5) expression was markedly reduced in all tissues, with minimal expression at the 5′- and 3′-ends of whirlin.

Brain, inner ear, and eye extracts were used to profile expression across whirlin (Figure 4), using several probes for 5 genotypes (*Whrn*^*+/+*^, *Whrn*^*hb/hb*^, *Whrn*^*hb/hb;BAC279+/−*^, *Whrn*^*hb/hb;BAC279−/-*^, and *Whrn*^*wi/wi*^). Wild-type expression of whirlin across these tissues was significantly higher (*p* < 0.05; ANOVA, Tukey post-hoc) within inner ears and eyes compared with brain (Table S6). The profile of expression downstream of the mutation site within each tissue showed that mutant transcripts were significantly reduced (*p* < 0.05; ANOVA, Tukey post-hoc) against wild type (Table S7). Transcription between exons 9–10 was broadly affected in *Whrn*^*hb/hb*^ mutants across all tissues while overexpression of the wild-type short-isoform (*Whrn*^*hb/hb;BAC279+−/−*^) significantly altered transcription levels only in the brain. Intriguingly, inner ear expression between exons 9–10 was markedly reduced while expression across exons 12–13 was comparable to wildtype levels, suggesting exon-skipping around the mutation site. Moreover, the transgene (*Whrn*^*hb/hb;BAC279+/−*^), showed minimal impact on expression across exons 9–10 within inner ears and eyes compared to mutants (*Whrn*^*hb/hb*^ and *Whrn*^*hb/hb;BAC279−/-*^). These results concur with phenotypic outcomes, where rescue of hyperactivity was observed in transgenic mutants, but moderate hearing loss remained unaltered. As expected, similar expression profiles were observed between *Whrn*^*hb/hb*^ and *Whrn*^*hb/hb;BAC279−/-*^ for each tissue and probe set. In comparison to all other genotypes, *Whrn*^*wi/wi*^ mutants showed significantly reduced whirlin expression spanning the deletion site (exons 6–12) across all tissues compared with each genotype. Expression of the intron retention transcript was also abundantly expressed in mutant genotypes against wild type for all tissues (data not shown). Transcripts upstream of the mutation were not significantly different between wild type and mutant genotypes across all tissues, excluding *Whrn*^*wi/wi*^.

Multiple comparisons using ANOVA and Tukey post-hoc analyses of ΔCT means within each tissue against genotype and whirlin probe confirmed observed increased expression was significant (*p* < 0.05; ANOVA, Tukey post-hoc) in brain tissue of BAC279-positive mice. Expression between tissues for each probe within a genotype was also found to be significantly (*p* < 0.05; ANOVA, Tukey post-hoc) different (Table S7).

Expression profiles of the 5 genes (*Akna*, *Orm2*, *Orm3*, *Orm1*, and *Col27a1*) contained within BAC279 were investigated (Figure S7; Table S7). Expression of these genes was distinctly increased in transgenics (*Whrn*^*hb/hb;BAC279+/−*^) across all tissues with the exception of *Col27a1*, which was solely upregulated in brain. Significant differences (*p* < 0.05; ANOVA, Tukey post-hoc) across ΔCT means were found between *Whrn*^*hb/hb;BAC279+/−*^ and each of the genotypes. *Orm2* and *Orm3* expression are lacking from *Whrn*^*wi/wi*^ mutants, supporting findings previously reported on the absence of these genes.^1^ Notably, no expression differences were identified for these genes between remaining genotypes (*Whrn*^*+/+*^, *Whrn*^*hb/hb*^, and *Whrn*^*hb/hb;BAC279−/-*^), suggesting baseline transcription of these genes inherently is not impacting phenotypes observed in mutants. In summary, we cannot rule out that overexpression of additional genes within BAC279 transgene affects the behavioral phenotype, but our data implicate significant overexpression in the transgenics of C-terminal whirlin isoform in the brain is driving observed changes in hyperactivity.

## Discussion

Broad-based phenotype screening is increasingly recognized as fundamental to a comprehensive understanding of wider gene function and uncovering pleiotropy.^24^^,^^38^^,^^39^ Our ENU aging-screen employing multiple phenotyping platforms identified a recessive SNV in whirlin resulting in mutants characterized with head-bobbing, hyperactivity, and mild-moderate hearing loss. The discovery and study of the *head-bob* mutant revealed a novel pleiotropic function for whirlin.

Sequenced transcripts over the SNV found a 25 bp intron retention at the donor splice site that caused a frameshift. A downstream PTC was introduced in the altered reading frame, which would be expected to result in NMD of the transcript. Lacking a whirlin antibody that could be used reliably to study protein function of the various isoforms, we used several qPCR probes across the gene and tissue extracts from brain, inner ears, and eyes in order to dissect the impact our mutation had on expression of transcripts. Expression profiling across the gene indicated that baseline expression of whirlin was predominant within inner ears and eyes with lower levels of expression in brain. Significantly reduced transcription, spanning the region over and downstream of the mutation, was found in all tissues compared to wild types. The presence of the BAC-transgene, spanning the C-terminal short isoform, only significantly upregulated transcripts in brain tissue and coincided with rescue of the hyperactive phenotype observed. A distinct reduction in expression of transcripts spanning exons 9–10 was found within inner ears, which subsequently increased to levels comparable to wildtype at the C-terminal PBM (PDZ-binding motif). 5′-donor splice site point mutations have been reported to activate cryptic splice sites either upstream or downstream whereby exon skipping and/or partial retention of intronic sequence can occur.^40^^,^^41^^,^^42^ We did not detect alternative splice forms that suggested a truncated transcript was produced, but exon 8 was skipped in all genotypes unless a primer was designed within it, as evidenced by sequencing. The retained intron splice-site boundary indicated features of a 5′ cryptic splice site (ATCCTTG/GUG sequence), which efficiently spliced into exon 9 where the PTC was located. A possible explanation for the profile of expression observed in the inner ears could be specific downregulation over the transcript containing the PTC that promotes and strengthens splicing further downstream to evade deleterious effects of the mutation and thereby NMD, as has been reported to occur at cryptic splice sites.^40^^,^^41^ Given the presence of the PBM at the C-terminal and the crucial role whirlin plays within the Usher interactome, evolutionary selection pressure of splicing regulation at the terminus of whirlin could aid in evading redundancy exclusively in the inner ears. We can presume from these data that *Whrn*^*hb/hb*^ transcripts underwent NMD in the brain and eyes based on reduced expression found. Moreover, the putative cryptic splice site affecting expression of exons 9–10 was only active within the inner ears.

Complementation testing of our mutation over the *whirler* mutation confirmed whirlin was the gene underpinning the *Whrn*^*hb/hb*^ phenotype. The corollary was that severity of deafness observed in *Whrn*^*hb/hb*^ mutant mice was mild-moderate in comparison to *Whrn*^*wi/wi*^ mutants despite the SNV being located within the *whirler* deletion site, in which N-terminal transcripts and potentially a spliced variant around the deletion site are reported to be retained.^5^^,^^6^ In addition, impaired hearing in *Whrn*^*hb/hb*^ mutant mice was much less acute than in knockout lines, which predominantly affect the N-terminal long isoform with predicted retention of C-terminal transcript.^5^^,^^6^ Differences in severity of deafness in our study could be driven by background strain effects. *Head-bob* mice carrying the BAC279 transgene (*Whrn*^*hb/hb;BAC279+/−*^) overexpressing wild-type C-terminal short isoform, did not have hearing levels paralleling *Whrn*^*+/+*^ mice but mild-moderate deafness similar to those recorded from *Whrn*^*hb/hb*^ mutants. These results validate previous reports on BAC279 transgenics, where it was shown that mice carrying the transgene over the *Whrn*^*wi/wi*^ mutation did not rescue hearing dysfunction.^5^ SEM of IHC and OHC stereocilia similarly showed minimal defects across *Whrn*^*hb/hb*^ mutants. OHC stereocilia bundles of *Whrn*^*hb/hb*^ were distinctly set apart and rounded in shape, particularly within medial and basal turns of the organ of Corti, comparable to previous studies on whirlin knockout mice.^1^^,^^5^^,^^6^ Our results therefore recapitulated the knockout phenotype rather than the phenotype displayed by deletion of the C-terminal short isoform. Electrophysiological results suggested a potential impairment in the Ca^2+^ sensitivity of the MET channels in mutant OHCs—a dominant effect requiring further investigation. Given mutant whirlin IHCs, particularly *Whrn*^*wi/wi*^, lack maturation of *I*_*k.n*_ and *I*_*k,f*_ currents, further highlights the requisite role of whirlin for the development of IHCs. ABR waveform analyses further demonstrated an increased amplitude and delayed latency of wave II for both *Whrn*^*hb/hb*^ and *Whrn*^*hb/hb;BAC279+/−*^ compared with *Whrn*^*+/+*^ mice, which indicated a qualitative difference in auditory transduction. By proxy of wave II,^43^ these waveform profiles suggested that our *Whrn*^*hb/hb*^ mutation had an intrinsic temporal processing deficit of the acoustic stimulus, with potential dysfunction of whirlin in the ascending auditory pathway to the thalamus. The increased amplitude of wave II observed in mutants could be explained by a simultaneous increased spontaneous activity in response to the acoustic stimulus that reduces tuning width and dynamic range, which aligns with the notion that temporal coding changes in the central auditory pathway are affected with inner ear dysfunction and hearing loss.^44^^,^^45^ These outcomes could partially underpin some of the phenotypes observed across our mutant. Impaired hearing could partly be explained by insufficient priming of sensory neurons to the auditory stimulus in our whirlin mutant, due to immature formation of these neurons in the central auditory pathway, as found in *whirler* mutants.^23^ Overall, our data confirm the indispensable role of whirlin for proper sensorineural hearing function and support its centrally mediated function in the brain.

Phenotyping in the HCA demonstrated marked hyperactivity in *Whrn*^*hb/hb*^ mice compared with *Whrn*^*+/+*^ littermates. This was notable at the onset and throughout the dark phase and transitioning into the light phase, leading to a distinct triphasic pattern of activity. PIR activity supported these data across the 12 h light:12 h dark cycle. Interestingly, when exposed to constant darkness, hyperactivity was much less pronounced and steadily attenuated in *Whrn*^*hb/hb*^ mice suggesting the phenotypic outcome could be potentiated by light. Investigating ONL thickness as an index of degeneration within the light-sensing part of the retina revealed no specific differences across genotypes. Mice carrying the BAC279 transgene had reduced ONL, which could reflect a modifier effect of the variable genetic background introduced from the transgenic line since it was originally derived using CBA/B6 oocytes. Studying the ONL phenotype on a genetically homogeneous background would merit further study to elucidate whether the variation observed is attributed to strain effects. It is likely however, that retinal defects had not yet developed in these mutants since previously these were observed between 28 and 33 months of age.^4^^,^^5^ Open field movement tracks of *Whrn*^*hb/hb*^ and *Whrn*^*hb/wi*^ mice revealed significantly increased hyperactivity across the arena with little distinction across zones, substantiating HCA and PIR results and indicated outcomes were not anxiogenic. Remarkably, addition of the transgene to mutants (*Whrn*^*hb/hb;BAC279+/−*^ and *Whrn*^*hb/wi;BAC279+/−*^) restored activity levels to those of *Whrn*^*+/+*^ and *Whrn*^*+/hb*^. This confirmed the role of whirlin in the observed hyperactive phenotype. Taken together, these data demonstrate a centrally mediated neurological and novel pleiotropic function for whirlin.

Vestibular defects could underpin hyperactivity in *Whrn*^*hb/hb*^ mutants given reported VsEP deficits and loss of saccular and utricular function across whirlin mutants.^16^ We found some stereotypic behaviors on disturbance of the home cage and hyperactivity around the cage including climbing rotations on the cage lid but minimal circling behaviors of *Whrn*^*hb/hb*^ mutants. Swim test (data not shown) did not reveal specific behaviors characteristic of overt vestibular defects. However, it is possible that some of the behaviors observed, and the head-bobbing phenotype relate to a macula organ deficit. Diminished hyperactivity in constant darkness, toward the end of the PIR screen, could potentially be attributed to a vestibular ocular reflex (VOR) deficit, a response mediated by the semi-circular canals, such that complete lack of light stimulus may have attenuated the VOR and reduced hyperactivity as a result. These results are supported by a study that assessed the integrity of sinusoidal rotation across *Pcdh15*^*av−3J*^ deaf-circling mutants in light versus dark, using varying frequencies of the optokinetic reflex, which identified an absent angular VOR in the dark and a reduced gain at higher frequencies of a visual stimulus.^46^ It was postulated that these outcomes were driven by lack of visual input during darkness and an upstream defect of the secondary vestibular neurons in either the semi-circular canals or primary vestibular neurons, which are mediated centrally. Our *Whrn*^*hb/hb*^ mutation could have resulted in a peripheral and centrally regulated vestibular deficit, which led to diminished VOR during constant darkness in PIR. Additional data implicating this pathway in our whirlin mutant comes from an investigation that generated a series of elegant crosses on *Tbx1*^*Cre/+;*^*Slc12a2*^*fx/fx*^ mice, which had profound sensorineural hearing loss and were mildly or severely hyperactive.^47^ They assessed macula organ function from VsEPs and found that mutant mice severely hyperactive around 6–8 weeks had no VsEP response. To elucidate the role the CNS played in integrating sensory information via the Scarpa’s ganglion, which branches out into the brainstem vestibular nuclei (VN), they used stereotaxic injection to lesion VN of *Rosa26*^*DTA/DTA*^ mice, conditionally expressing diphtheria toxin A, with AAV1-Cre virus. Using NeuN staining, they found a significant loss of neurons within medial VN that extends into the thalamus, as well as a proportional loss of neurons in the lateral, superior, and inferior subnuclei of mice specifically targeted for lesion of VN. Ten days post-injection, a hyperactive response was observed in mice with successfully lesioned VN that was purported to affect both peripheral and central mediated vestibular dysfunction. Considering interactions of VN with the thalamus, which serves as a conduit to relaying and modulating sensorineural information in the brain^48^ along with previous findings on the impact of whirlin in proper compaction at paranodal junctions,^20^ it is tempting to infer that our mutation disrupts the vestibulothalamic pathway and rescue of hyperactivity in *Whrn*^*hb/hb;BAC279+/−*^ mice overexpressing the C-terminal isoform could be explained by its upregulation within neurons of the reticular pathway. Overall, these results substantiate a functional role for whirlin in the brain since mutational loss of function was shown to drive hyperactivity. Furthermore, it has wider implications of the utility that the brain has on the prevalence of comorbid hearing loss associated with hyperactive disorders.^49^^,^^50^

In summary, we have shown that a novel donor-splice site SNV in whirlin generated in our ENU-mutagenesis screen led to hyperactivity, which was rescued by a BAC transgene expressing the C-terminal region of the gene. Bringing together the results presented here and from previous studies the data infers a role for whirlin in the vestibulothalamic pathway that accounts for the hyperactive behaviors in *hb* mutants. Moreover, the findings give further weight to a centrally mediated function for whirlin in the brain. In addition, despite the modest hearing deficit observed in *hb* mutants, differences were observed in the structure and distribution of OHC stereocilia bundles, and temporal processing of the acoustic stimulus was impaired, potentially stemming from deficits in the central auditory pathway. Overall, the findings reported here could have relevance for our understanding of hyperactive disorders associated with hearing loss.

### Limitations of the study

The vestibular system was not broadly characterized to confirm the upstream impact of the mutation on the trajectory of neurons within the vestibulothalamic pathway. Evidence from VsEPs across other whirlin mutants revealed an impaired response from sound-induced evoked potentials indicating a direct loss of saccular and utricular function^17^ and we might expect a similar phenotyping profile in our *Whrn*^*hb/hb*^ mutant. However, to fully elucidate the deeper mechanisms of the hyperactive response observed with the *Whrn*^*hb/hb*^ mutation on the vestibulothalamic pathway and its interactions with the somatomotor cortex, we could utilize an *in vivo* tool that enables tracing of neuronal activity at the cellular level during behavioral screening. A recent advance that has been developed and uses a calcium sensor to capture single-cell resolution cortical activity called CaMPARI^51^ during behavioral testing in mice, could demonstrate how the potential loss of calcium sensitivity, which was found from MET currents in our study, is more widely impacted across neural circuitry in the brain of the *Whrn*^*hb/hb*^ mutants. Direct mapping of the neural networks during free moving activity would enable us to identify and confirm the involvement of the specific pathways that underpin the hyperactive response of *Whrn*^*hb/hb*^ mutant mice.

## STAR★Methods

### Key resources table

**Table:** 

REAGENT or RESOURCE	SOURCE	IDENTIFIER
**Critical commercial assays**		

High-Capacity cDNA Reverse Transcription kit	ThermoFisher Scientific	4374966
Maxwell® 16 Tissue LEV Total RNA Purification Kit	Promega	AS1225
Phusion® High-Fidelity DNA Polymerase	New England Biolabs Ltd	M0530
Fast SYBR® Green Master Mix	ThermoFisher Scientific, UK	10459604
TaqMan™ Fast Universal PCR Master Mix (2X)	ThermoFisher Scientific, UK	10637755
TaqMan™ assay (*Col27a1*)	ThermoFisher Scientific	Mm00508542_m1
TaqMan™ assay (*Orm1*)	ThermoFisher Scientific	Mm00435456_g1
TaqMan™ assay (*Orm2*)	ThermoFisher Scientific	Mm04213463_g1
TaqMan™ assay (*Orm3*)	ThermoFisher Scientific	Mm03010552_g1
TaqMan™ assay (*Akna*)	ThermoFisher Scientific	Mm01258100_m1
TaqMan™ assay (*Gapdh*)	ThermoFisher Scientific	Mm99999915_g1
TaqMan™ assay (*Ppia*)	ThermoFisher Scientific	Mm02342429_g1
TaqMan™ assay (*Actb*)	ThermoFisher Scientific	Mm02619580_g1

**Deposited data**		

Whole Genome Sequencing data	NCBI Short Read Archive database, as listed in Potter et al.^24^	Database: https://www.ncbi.nlm.nih.gov/Traces/sra/sra.cgi? Accession number PRJNA322401; Individual SRA biosample ID: MPC-91, SAMN05172946
Raw data output files including metadata for ABR, HCA, OCT, open field, PIR, qPCR and electrophysiology data	This paper, Mendeley Data	Mendeley Data: https://data.mendeley.com/datasets/9xw6fc9j54/1
Original R code generated for phenotyping analysis and qPCR	This paper, Mendeley Data	Mendeley Data: https://data.mendeley.com/datasets/9xw6fc9j54/1

**Experimental models: Organisms/strains**		

C3.C-Pde6b+/4H^52^	https://www.informatics.jax.org/strain/summary?strainName=C3.C-Pde6b%3C%2B%3E%2F4H&attributeOperator=any	RRID: MGI:5505790
*Whrn*^*hb/hb*^	https://www.har.mrc.ac.uk	RRID: IMSR_HAR:9372
*Whrn*^*hb/wi*^	https://www.har.mrc.ac.uk	RRID: IMSR_HAR:7803
*Whrn*^*hb/hb;BAC279+/-*^	https://www.har.mrc.ac.uk	RRID: IMSR_HAR:8061
*Whrn*^*hb/wi;BAC279+/-*^	https://www.har.mrc.ac.uk	RRID: IMSR_HAR:7804
*Whrn*^*wi/wi;BAC279+/-*^ also known as Tg(Whrn)#Ptt	https://www.har.mrc.ac.uk	RRID: MGI:5616436
*Whrn*^*wi/wi*^	https://www.har.mrc.ac.uk	RRID: MGI:1857090

**Oligonucleotides**		

Primer (forward) flanking splice site: GACTCGTGCACTGCTGGACG	This paper	N/A
Primer (reverse) flanking splice site: CCTGTGC TGAGGAAGGTCCCG	This paper	N/A
Forward TaqMan™ probe for genotyping *Whrn*^*+/+*^ allele: GGGTTGGAGACACGTATTCCA	This paper	N/A
Reverse TaqMan™ probe for genotyping *Whrn*^*+/+*^ allele: GGCAGGGGTGGCTCAC	This paper	N/A
Forward TaqMan™ probe for genotyping *Whrn*^*hb/hb*^ SNV: CGTCAGCTCGGCAAGGA	This paper	N/A
Reverse TaqMan™ probe for genotyping *Whrn*^*hb/hb*^ SNV: CCGAGGCTTAGGAGGGAAAC	This paper	N/A
FAM labelled probe for *Whrn*^*+/+*^: CAGCTCGGCC AGGGTG	This paper	N/A
FAM labelled probe for *Whrn*^*hb/hb*^: CCCTGCCCT ATCCTTGGTGAGAA	This paper	N/A
Forward TaqMan™ probe for genotyping *Whrn*^*wi/wi*^ allele: GCCTAGATCCACCCTCTCATAAC	This paper	N/A
Reverse TaqMan™ probe for genotyping *Whrn*^*wi/wi*^ allele: TCCCGAGACTGTAGCTCAGTA	This paper	N/A
FAM labelled probe for *Whrn*^*wi/wi*^: TCAGGCGGTGA ATAGATTATTTGCATAGGT	This paper	N/A
Forward TaqMan™ probe for genotyping CMR of the BAC: GCCCCAGCACGACCATT	This paper	N/A
Reverse TaqMan™ probe for genotyping CMR of the BAC: TAGTTGGCATCCTTATGCTTCATC	This paper	N/A
FAM labelled probe for CMR of the BAC: CCAGCTC TCAAGTCG	This paper	N/A
Primer (forward) *Whrn2-3*: CGTGAAGATGACCGAA GGAGTAC	This paper	N/A
Primer (reverse) *Whrn2-3*: GGCCGTCCCCCAACAC	This paper	N/A
Primer (forward) *Whrn6-7*: TCATGGCACTGTTTG AGTTGCT	This paper	N/A
Primer (reverse) *Whrn6-7:* GATGATGCTTCTCACC TCAGACA	This paper	N/A
Primer (forward) *Whrn7-intret*: TGACCACCTGGT GCTGAGGC	This paper	N/A
Primer (reverse) *Whrn7-intret:* AAGGATAGGGCAGGGGTGGCTCAT	This paper	N/A
Primer (forward) *Whrn9-10*: CGCTCTCCCGGATGTGTCT	This paper	N/A
Primer (reverse) *Whrn9-10:* CTTGATACCAGGCAGATCTTCTGA	This paper	N/A
Primer (forward) *Whrn12-13*: ATCGTCACAATTCAGCGAGG	This paper	N/A
Primer (reverse) *Whrn12-13:* CTAGAGCATCACGTTGAACTC	This paper	N/A
Primer (forward) *Gapdh*: CGGCCGCATCTTCTTGTG	This paper	N/A
Primer (reverse) *Gapdh:* CCGACCTTCACCATTTTGTCTA	This paper	N/A
Primer (forward) *Actinb*: CGATGCCCTGAGGCTCTTT	This paper	N/A
Primer (reverse) *Actinb:* TGGATGCCACAGGATTCCAT	This paper	N/A
Primer (forward) *Ppia*: AGTTTTTTATCTGCACTGCCAAGA	This paper	N/A
Primer (reverse) *Ppia:* CCTTCCCAAAGACCACATGCT	This paper	N/A

**Software and algorithms**		

Actual Analytics	Bains et al.^53^	https://www.actualanalytics.com
COMPASS system	Brown et al.^54^	https://wellcomeopenresearch.org/articles/1-2/v2
Ethovision XT software	Noldus, Wageningen, The Netherlands	https://www.noldus.com/ethovision-xt
BioSig software	Tucker Davies Technology, USA	https://biosig.sourceforge.net
pClamp software	Molecular Devices, USA	https://www.moleculardevices.com/sites/default/files/en/assets/user-guide/dd/cns/digidata-1440a-low-noise-data-acquisition-system.pdf
Origin software	OriginLab, USA	https://www.originlab.com/index.aspx?go=Products/Origin#Data_Analysis_and_Statistics
R Statistical Software	v4.3.2, 2023-10-31	https://www.r-project.org
GraphPad 9.0	Dotmatics, USA	https://www.graphpad.com
7500 Real-Time PCR Software v2.0.6	Applied Biosystems®, UK	https://www.thermofisher.com/fr/fr/home/technical-resources/software-downloads/applied-biosystems-7500-real-time-pcr-system.html
R Statistical Software	v4.3.2, 2023-10-31	https://www.r-project.org

**Other**		

TDT System 3	Tucker Davies Technology	https://www.tdt.com/systems/auditory-research-systems/
Scanning Electron Microscope	JEOL	https://www.jeol.com/products/scientific/sem/JSM-6010LA.php
OptoPatch amplifier	Cairn Research	https://www.cairn-research.co.uk/products/
Bioptigen Envisu™ SDOCT R2200	Leica Microsystems	https://www.leica-microsystems.com/products/surgical-microscopes/p/envisu-r-class/
Digidata 1440A boards	Molecular Devices	https://www.moleculardevices.com/sites/default/files/en/assets/user-guide/dd/cns/digidata-1440a-low-noise-data-acquisition-system.pdf

### Resource availability

#### Lead contact

Further information and requests for resources and reagents should be directed to and will be fulfilled by the lead contact, Dr Heena V. Lad (hvlad10@hotmail.com).

#### Materials availability

Mouse strains in this study were generated in the Mary Lyon Centre at MRC Harwell and can be provided pending a completed material transfer agreement from the MRC National Mouse Archive repository. All RRID numbers can be found in the key resources table.

#### Data and code availability

• Output raw data files generated from each phenotyping test (HCA, PIR, open field, ABR, and OCT), qPCR experimentation and electrophysiology results, including metadata information, are publicly available in a Mendeley Data repository and is publicly available as of the date of publication. DOIs are listed in the key resources table.
• All original R code generated for phenotyping analysis, has been deposited in a Mendeley Data repository and is publicly available as of the date of publication. DOIs are listed in the key resources table.
• Whole genome sequencing data have been deposited at NCBI Short Read Archive database, as listed in Potter et al.,^24^ and are publicly available as of the date of publication. The Database and Accession number is listed in the key resources table.
• Any additional information required to reanalyse the data reported in this work paper is available from the lead contact upon request.

### Experimental model and study participant details

All mice (*Mus musculus*) were maintained, and procedures carried out in compliance with Animals (Scientific Procedures) Act 1986, UK, Amendment Regulations 2012 (SI 4 2012/3039) and the Ethical Review Committee at MRC Harwell and the University of Sheffield Ethical Review Committee (Responsibility in the Use of Animals for Medical Research, July 1993; Home Office licences 30/3070, 30/3206 and PCC8E5E93). Mice bred at the Mary Lyon Centre (MLC) were group housed (≥3-5 per cage) in IVCs (Individually Ventilated Cages; Tecniplast BlueLine 1284), of mixed genotypes pseudorandomised at weaning. Access to a commercial diet (SDS Rat and Mouse No. 3 Breeding diet, RM3) and water (25 p.p.m chlorine) was available *ad libitum*. Lighting conditions within the animal house was maintained (07:00-19:00 light, 19:00-07:00 dark; with a ramp up/down time of 30 minutes at lights on/off, respectively), with temperature (21±2°C) and humidity (55±10%) ranges monitored. Health checks were performed once a week. Mice were maintained under comparable conditions at Sheffield University.

Phenotyping procedures were carried out in age-matched, adult mice (from ≥8 weeks) and genotyping groups balanced for males and females (n≥7 per sex) in all tests, excluding ABR and OCT where reduced numbers were sufficient to detect an effect. Electrophysiology recordings were undertaken between postnatal day (P) P5-32 and did not differentiate sex effects. Expression profiling was performed on P5-6 animals and did not account for sex differences wherein a minimum of 5 mice per genotype used. Weights were routinely measured and recorded on a laboratory information management system (LIMS), and before some of the phenotyping procedures. Main effects were observed by genotype with minor sex differences identified and reported from statistical analyses.

The *Whrn*^*hb/hb*^ ENU mutant (RRID: IMSR_HAR:9372) was originally identified from The Harwell Ageing Screen.^24^ Mutant mice in this study were outcrossed to sighted C3H (C3H.Pde6b+,^52^ RRID: MGI:5505790) repaired for the retinal degeneration allele and, genotyped to exclude propensity to hearing loss (*Cdh23*^*ahl*^), then maintained through backcrossing (>5 generations) to obtain a genetically homogeneous background. Compound heterozygotes were generated by initially outcrossing *Whrn*^*hb/hb*^ and *Whrn*^*wi/wi*^ mutants^1^ (RRID: MGI:1857090) and phenotyping cohorts produced by inter-crossing to obtain *Whrn*^*hb/wi*^ (RRID: IMSR_HAR:7803). BAC279 transgenic mice (RRID: MGI:5616436) were generated as previously reported^1^ and rederived in the MLC at Harwell, which were outcrossed to *Whrn*^*hb/hb*^ to generate transgenic animals overexpressing wildtype C-terminus whirlin over the *hb* allele and *whirler* deletion (*Whrn*^*hb/hb;BAC279+/-*^, RRID: IMSR_HAR:8061; *Whrn*^*hb/wi;BAC279+/-*^, RRID: IMSR_HAR:7804). Phenotyping was performed blind to genotype where possible.

### Method details

#### Mapping, mutation identification and validation

The mutation was mapped using DNA extracts from ear biopsies of affected and unaffected G3 mice and the GoldenGate Mouse Medium Density Linkage Panel (Gen-Probe Life Sciences Ltd, UK), which discriminated 900 single-nucleotide polymorphisms (SNPs) between the C3H.Pde6b+ and C57BL/6J strains.^24^ Whole-genome sequencing (WGS) from DNA extracts (Illustra Nucleon BACC2 Genomic DNA Extraction Kits, GE Healthcare Life Sciences) of the G1 founder male was undertaken using the Illumina HiSeq platform (Oxford Genomics Centre, Wellcome Trust Centre for Human Genetics). Analysis of WGS is described and previously reported.^24^ cDNA was synthesised (High-Capacity cDNA Reverse Transcription kit, ThermoFisher Scientific) from RNA extracts (Maxwell® 16 Tissue LEV Total RNA Purification Kit, Promega) to validate the SNV from brain, inner ears and eye tissue of wildtype and homozygote mice. Primers flanking the splice variant (5’-GAC-TCG-TGC-ACT-GCT-GGA-CG-3’ and 5’-CCT-GTG-CTG-AGG-AAG-GTC-CCG -3’) were used to amplify (Phusion® High-Fidelity DNA Polymerase, New England Biolabs Ltd, UK) and sequence across the SNV. Sequencing reactions were outsourced to Source BioScience, UK.

#### Genotyping

Copy number variant qPCR assays were used to genotype wildtype and mutant alleles from ear/tail biopsies DNA extracts and TaqMan™ probes (Applied Biosystems, UK). The G>A variant SNV at the splice site was differentiated with two sets of primer pairs for the wildtype (5’-GGG-TTG-GAG-ACA-CGT-ATT-CCA-3’ and 5’-GGC-AGG-GGT-GGC-TCA-C-3’) and mutant alleles (5’-CGT-CAG-CTC-GGC-AAG-GA-3’ and 5’-CCG-AGG-CTT-AGG-AGG-GAA-AC-3’) along with FAM labelled probes (5’-CAG-CTC-GGC-CAG-GGT-G-3’ and 5’-CCC-TGC-CCT-ATC-CTT-GGT-GAG-AA-3’). Samples were genotyped using both assays. The wildtype assay produced 2 copies for wildtype alleles, heterozygotes 1 copy and mutant allele would drop to 0; the inverse applied to the mutant assay. The *whirler* deletion genotyping used primers designed within the deletion site (5’-GCC-TAG-ATC-CAC-CCT-CTC-ATA-AC-3’ and 5’-TCC-CGA-GAC-TGT-AGC-TCA-GTA-3’) and a FAM probe (5’-TCA-GGC-GGT-GAA-TAG-ATT-ATT-TGC-ATA-GGT-3’). Primers located in the chloramphenicol (CMR) arms of the BAC (5’-GCC-CGC-CTG-ATG-AAT-GCT-3’ and 5’-CGG-TGT-AAC-AAG-GGT-GAA-CAC-TA-3’) and a FAM probe (5’-TCC-GGA-GTT-CCG-TAT-GGC-AAT-GAA-3’) were used to detect the presence of the transgene.

#### Phenotyping

Phenotype testing was carried out between 08:00-13:00 using male and female mice and, a representative number of each sex and genotype in accordance with the test paradigm. HCA and PIR were carried out over several days and used separate cohorts of animals. Mice were acclimatised to the test room for a minimum of 30 min before behavioural phenotyping testing commenced except for HCA, when 24 hours acclimation was given. All test equipment was wiped clean with 70% ethanol or 2% Distel between subjects and tests.

#### Home cage analysis (HCA)

Mice were group-housed (n=3 per cage) in IVC cages of the same sex and genotype. An RFID microchip (11.5 × 2 mm, PeddyMark Ltd. UK) was inserted subcutaneously in each mouse under sedation (Isoflo, Abbott, UK) and local anaesthetic (EMLA Cream 5%, AstraZeneca, UK) at 12 weeks of age, and given a minimum recovery period of a week before testing. Mice were placed and monitored in the HCA system (Actual Analytics, Edinburgh, UK) from 15:00 day0 for 3 days on a 12h light:12h dark cycle. Behaviour was monitored using both video tracking and location tracking of RFID-captured coordinates.^53^ ANOVA for each hourly bin regressed against genotype, sex and genotype by sex interaction calculated to determine significant differences (*p*<0.05).

#### Passive infrared (PIR)

Single-housed home cage activity was measured using passive infrared with the COMPASS system.^54^ Mice were individually housed, and data captured for 5 days in a 12h:12h light/dark cycle, followed by 9 days in constant darkness. Data analysis was extracted using custom written python scripts and macros. ANOVA for each hourly bin regressed against genotype, sex and genotype by sex interaction calculated to determine significant differences (*p*<0.05) during light/dark and constant darkness.

#### Open field

Activities in response to a novel environment were assessed using open field. Mice were placed individually into the corner of a square (44×44 cm), custom made opaque arena, within an enclosed test room. A minimum of 2 and maximum of 4 mice were tested per trial. Lighting was set at 150-200 lux in the centre of the arena. Behaviours were video tracked (20 minutes) and data analysed using Ethovision XT software (Noldus, Wageningen, The Netherlands) for distance moved, mean velocity and duration in pre-defined zones (e.g., central, peripheral, total) in the arena. Note that due ethical considerations *Whrn*^*wi/wi*^ mice could not be tested for activity-related tests. Tukey post-hoc multiple comparisons analysis using ANOVA of the parameter within each specific zone and bin against each genotype was calculated to determine significant differences (*p*<0.05).

#### Optical coherence tomography (OCT)

Mice were anaesthetised by intraperitoneal injection of ketamine (100 mg/ml at 10% v/v) and xylazine (20 mg/ml at 5% v/v) administered at 0.01 ml/g body mass. Eyes were dilated with tropicamide 1% and, eye lubricant applied (0.2 mg/g carbomer; Viscotears®, Novartis AG) to circumvent eyes drying out during the procedure. OCT images were captured with the Bioptigen Envisu™ SDOCT R2200 (Leica Microsystems, Germany). Six measures were obtained across six quadrants of the retina in the outer nuclear layer (ONL), and retinal layer thickness was averaged for each ONL measure per eye (left/right). Tukey post-hoc multiple comparisons analysis using ANOVA of each eye against genotype determined retinal layer thickness differences (p<0.05).

#### Auditory phenotyping

Auditory Brainstem Response (ABR) tests were performed using a click stimulus and frequency-specific tone-burst stimuli (8, 16 and 32 kHz) to investigate auditory function as described in.^55^ Mice were anaesthetised by intraperitoneal injection of ketamine (100 mg/ml at 10% v/v) and xylazine (20 mg/ml at 5% v/v) administered at 0.01 ml/g body mass and placed on a heated mat inside a sound-attenuated chamber (ETS Lindgren, USA). F-E2-12 recording electrodes (Grass Telefactor, USA) were placed sub-dermally over the vertex (active), right mastoid (reference) and left mastoid (ground). ABR traces were collected, amplified, and averaged using TDT System 3 hardware and BioSig software (Tucker Davies Technology, USA). Click stimulus consisted of a 0.1 ms broadband click presented at a rate of 21.1 s^-1^. Tone-burst stimuli were of 7 ms duration including rise/fall gating using a 1 ms Cos^2^ filter, presented at a rate of 42.5 s^-1^. All stimuli were presented free field to the right ear of the mouse, starting at 90 dB SPL and decreasing in 5 dB steps until a threshold was determined visually by the absence of replicable response peaks. Mice were recovered using 0.1 ml of anaesthetic reversal agent atipamezole (5 mg/ml at 1% v/v). Thresholds were compared using Tukey post-hoc multiple comparisons ANOVA. Waveform analyses were conducted with data extracted from 16 kHz traces, corrected for noise, adjusted to level over threshold and regressed to second-polynomial functions. An extra sum-of-squared F test with FDR<0.05 was used to determine if data from different experimental groups could be statistically fit into the same regression.

#### Scanning electron microscopy

Inner ears were dissected out of 1-month old mice, cleared of extraneous tissue and, nicked at the apex of the cochlea before fixing in freshly prepared 2.5% glutaraldehyde in 0.1 M sodium phosphate buffer (Sigma-Aldrich) for 3-4 hrs at 4°C.^56^ Samples were washed and decalcified in 4.3% EDTA for 48 h at 4°C. Inner ears were fine dissected and processed using alternate O_s_O_4_ (Agar Scientific, UK) and thiocarbohydrazide (Fluka, Sigma-Aldrich, UK), to preserve stereocilia structure. Dehydration of samples performed by immersion in ethanol solutions of increasing strength and stored in acetone until critical point drying. Mounted samples were sputter coated using platinum (Quorum Q150T, Quorum Technologies, UK) and imaged on a JEOL JSM 6010LV (Jeol Ltd, Japan).

#### Single-hair cell electrophysiology

Animals used for electrophysiological studies were licenced by the Home Office (PCC8E5E93) under the Animals (Scientific Procedures) Act 1986, UK, and approved by the University of Sheffield Ethical Review Committee. All recordings were performed at room temperature (22-24°C), excluding membrane capacitance.

Apical-coil OHCs and IHCs from *Whrn*^*+/+*^, *Whrn*^*hb/hb*^ and *Whrn*^*wi/wi*^ mice were studied in acutely dissected cochleae taken between P5-P32. Cochleae were dissected in normal extracellular solution (mM): 135 NaCl, 5.8 KCl, 1.3 CaCl_2_, 0.9 MgCl_2_, 0.7 NaH_2_PO_4_, 5.6 D-glucose, 10 HEPES-NaOH. Sodium pyruvate (2 mM), MEM amino acids solution (50X, without L-Glutamine) and MEM vitamins solution (100X) were added from concentrates (Fisher Scientific, UK) and, adjusted to pH 7.5 (308 mOsmol kg^-1^). Dissected cochleae were transferred to a microscope chamber, immobilised with a nylon mesh^25^^,^^57^ and, continuously perfused with a peristaltic pump using the normal extracellular solution. The organ of Corti were viewed using an upright microscope (Leica DMLMF, Germany; Nikon FN1, Japan) with Nomarski Differential Interface Contrast (DIC) optics (60x or 63x objectives). Patch clamp recordings were performed using an Optopatch amplifier (Cairn Research Ltd, UK). Patch pipettes were made from soda glass capillaries with a typical resistance in the extracellular solution of 2-3 MΩ. Patch electrodes were coated with surf wax (Mr Zoggs SexWax, USA) to reduce electrode capacitance.

Basolateral membrane ion currents were performed using an intracellular solution containing (mM): 131 KCl, 3 MgCl_2_, 1 EGTA-KOH, 5 Na_2_ATP, 5 HEPES-KOH, 10 Na_2_-phosphocreatine (pH 7.3; osmolality ∼296 mmol kg^-1^). Data acquisition was controlled by pClamp software using Digidata 1440A boards (Molecular Devices, USA). Recordings were low-pass filtered at 2.5 kHz (8-pole Bessel), sampled at 5 kHz, and stored on computer for off-line analysis (Origin, OriginLab, USA). Membrane potentials in voltage clamp were corrected for voltage drop across uncompensated residual series resistance (*R*_*s*_) and for a liquid junction potential (LJP: -4 mV). When investigating basolateral membrane properties, size of *I*_K,f_ was measured at 1 ms after stimulus onset at a membrane potential of -25 mV,^58^ while *I*_K,n_ was measured as the difference between the peak and steady state of deactivating inward current at -124/-144 mV, starting at a holding potential of -84/-64 mV (OHC/IHC, respectively).^29^ Steady-state total currents were measured at 160 ms, and potential of 0 mV.

Mechano-electrical transducer (MET) currents elicited by stimulating stereocilia bundles of OHCs, using a fluid jet from a pipette (tip diameter 8-10 μm) driven by a piezoelectric disc.^26^^,^^28^ The pipette tip of the fluid jet was positioned next to bundles to elicit a maximal MET current. Mechanical stimuli were applied as 50 Hz sinusoids (filtered at 0.25 kHz, 8-pole Bessel) with driving voltages of ± 40 V. MET currents recorded with a patch pipette solution containing (mM): 106 Cs-glutamate, 20 CsCl, 3 MgCl_2_, 1 EGTA-CsOH, 5 Na_2_ATP, 0.3 Na_2_GTP, 5 HEPES-CsOH, 10 sodium phosphocreatine (pH 7.3). Membrane potentials were corrected for using an LJP of -11 mV.

Real-time changes in membrane capacitance (*ΔC*_*m*_) performed at body temperature as previously described.^31^^,^^35^ A 4 kHz sine wave of 13 mV RMS applied to IHCs from -81 mV and interrupted during the voltage step. Capacitance signal from the Optopatch filtered at 250 Hz and sampled at 5 kHz. *ΔC*_*m*_ was measured by averaging *C*_*m*_ trace over a 200 ms period after the voltage step and subtracting the pre-pulse baseline. Membrane potentials were corrected for the voltage drop across the residual *R*_s_ and an LJP of -11 mV. *ΔC*_*m*_ experiments were performed in the presence of 30 mM TEA, 15 mM 4-AP (Fluka, Sigma-Aldrich, UK) and 80 μM Linopirdine (Tocris, UK) to block majority of K^+^ currents in hair cells.^29^^,^^32^^,^^58^

Statistical comparisons of means were made by the two-tailed *t-*test or for multiple comparisons, analysis of variance (one-way ANOVA and Tukey post-hoc). Means reported ± S.E.M. and *P*< 0.05 statistical significance.

#### qPCR

Tissues were harvested from mice aged P5-6 and snap frozen. RNA was extracted with the Maxwell 16 Instrument (Maxwell® 16 Tissue LEV Total RNA Purification Kit, Promega, UK). RNA was quantified (NanoDrop™ 8000 spectrophotometer, ThermoFisher Scientific, UK), and qualitatively assessed (Agilent RNA Nano kit, UK; 2100 Bioanalyzer instrument). cDNA was synthesised using 1-2ug of RNA (High-Capacity cDNA Reverse Transcription kit, ThermoFisher Scientific, UK). qPCR reactions were performed in triplicate on a 7500-fast machine (ThermoFisher Scientific, UK). Primers pairs were designed in house for SYBR® Green assays (*Whrn2-3*: 5’CGT GAA GAT GAC CGA AGG AGT AC-3’, 5’-GGC CGT CCC CCA ACA C-3’; *Whrn6-7*: 5’-TCA TGG CAC TGT TTG AGT TGC T-3’, 5’-GAT GAT GCT TCT CAC CTC AGA CA-3’; *Whrn7-intret (intron retention)*: 5’- TGA CCA CCT GGT GCT GAG GC-3’, 5’- AAG GAT AGG GCA GGG GTG GCT CAT-3’; *Whrn9-10*: 5’-CGC TCT CCC GGA TGT GTC T-3’, 5’-CTT GAT ACC AGG CAG ATC TTC TGA-3’; *Whrn12-13*: 5’-ATC GTC ACA ATT CAG CGA GG-3’, 5’-CTA GAG CAT CAC GTT GAA CTC-3’; *Gapdh*: 5’-CGG CCG CAT CTT CTT GTG-3’, 5’-CCG ACC TTC ACC ATT TTG TCT A-3’; *Actinb*: 5’-CGA TGC CCT GAG GCT CTT T-3’, 5’-TGG ATG CCA CAG GAT TCC AT-3’; *Ppia*: 5’-AGT TTT TTA TCT GCA CTG CCA AGA-3’, 5’-CCT TCC CAA AGA CCA CAT GCT-3’) and processed with Fast SYBR® Green Master Mix (ThermoFisher Scientific, UK). Custom primers for TaqMan™ reactions (*Col2741* Mm00508542_m1; *Orm1* Mm00435456_g1; *Orm2* Mm04213463_g1; *Orm3* Mm03010552_g1, *Akna* Mm01258100_m1; *Gapdh* Mm99999915_g1, *Ppia* Mm02342429_g1, *Actb* Mm02619580_g1) used Fast Universal PCR Master Mix (2X) (ThermoFisher Scientific, UK). All primers were validated for amplification efficiency and endogenous controls determined dependent on tissue type. Fold-changes calculated using the 2-ΔΔCt method using 7500 Real-Time PCR Software v2.0.6 (Applied Biosystems®, UK). Tukey post-hoc multiple comparisons analysis using ANOVA of ΔCT means against each genotype per gene expression probe and sample type was calculated to determine qPCR expression differences (*P*<0.05).

### Quantification and statistical analysis

Statistical analyses for HCA, PIR, open field, ABR, OCT and qPCR tests were performed using R Statistical Software (v4.3.2, 2023-10-31) to calculate significance, defined as *P* <0.05. Note that statistical analyses were calculated independently for each genotype, sex, and genotype by sex interactions and are reported in the tables within the supplemental information. Statistical analysis of ABR waveform was performed with GraphPad 9.0 (Dotmatics, USA). Details of the specific statistical tests performed for each test can be found within the respective method details. Number of animals (n) is represented in behavioural phenotyping figures and indicated within each figure legend and represent data by genotype. Given the minor effect of sex on phenotyping outcomes (see supplemental information), data are illustrated as means per genotype for each measure and, where error bars are plotted, represent either +/- confidence interval width (95%) (open field, ABR and OCT) or, +/- standard error of the mean (SEM; ABR waveform and electrophysiology). In electrophysiology data exclusively, found within supplemental information, n represents the number OHCs/IHCs recorded and is indicated within both the corresponding main results text, figures, and the respective legends. Data analysis for electrophysiology was performed with Origin software (OriginLab, USA).
