# Supplementary material for: Pleiotropic brain function of whirlin identified by a novel mutation
Source: iScience. 2024 Jun 4;27(7):110170. doi: 10.1016/j.isci.2024.110170 (PMC11225360; doi:10.1016/j.isci.2024.110170)
Supplement: Document S1. Figures S1–S7 and Tables S1–S8 [file mmc1.pdf]

iScience, Volume *27*

Supplemental information

Pleiotropic brain function of whirlin identified by a novel mutation

Carlos Aguilar, Debbie Williams, Ramakrishna Kurapati, Rasneer S. Bains, Philomena Mburu, Andy Parker, Jackie Williams, Danilo Concas, Hilda Tateossian, Andrew R. Haynes, Gareth Banks, Pratik Vikhe, Ines Heise, Marie Hutchison, Gemma Atkins, Simon Gillard, Becky Starbuck, Simona Oliveri, Andrew Blake, Siddharth Sethi, Saumya Kumar, Tanaya Bardhan, Jing-Yi Jeng, Stuart L. Johnson, Lara F. Corns, Walter Marcotti, Michelle Simon, Sara Wells, Paul K. Potter, and Heena V. Lad

Supplementary Figures and Tables

Table S1. HCA *P*-values using ANOVA for each hourly bin in a linear model regressed against genotype and sex and genotype x sex interaction related to Figure 2A.

Homecage bins (day_hour)	*Genotype*	*Sex*	*Genotype x Sex*
day0_15	0.248747	0.176017	0.664291
day0_16	0.484688	0.157592	0.229542
day0_17	0.172190	0.009874	0.558755
day0_18	0.039042	0.655371	0.813038
day0_19	0.034029	0.436083	0.666469
day0_20	0.037783	0.341505	0.625374
day0_21	0.050686	0.445533	0.748167
day0_22	0.029670	0.048077	0.421440
day0_23	0.043099	0.582655	0.940976
day1_0	0.024867	0.610052	0.996007
day1_1	0.016478	0.645257	0.612544
day1_2	0.075875	0.563834	0.209932
day1_3	0.244644	0.416537	0.022832
day1_4	0.215438	0.810246	0.097954
day1_5	0.288008	0.310985	0.709652
day1_6	0.107464	0.820696	0.436191
day1_7	0.053257	0.506793	0.904085
day1_8	0.048945	0.464292	0.653845
day1_9	0.064349	0.667076	0.634203
day1_10	0.071702	0.223355	0.244999
day1_11	0.074336	0.145508	0.002311
day1_12	0.180059	0.185520	0.165583
day1_13	0.155830	0.153456	0.163723
day1_14	0.311169	0.041361	0.129156
day1_15	0.592709	0.056135	0.012045
day1_16	0.255986	0.078696	0.216720
day1_17	0.244743	0.016894	0.296344
day1_18	0.067325	0.245746	0.534844
day1_19	0.015557	0.378672	0.515653
day1_20	0.009707	0.506238	0.734907
day1_21	0.018871	0.859205	0.974929
day1_22	0.034380	0.760021	0.955560
day1_23	0.023081	0.470620	0.778352
day2_0	0.025563	0.408786	0.519948
day2_1	0.022895	0.525940	0.617909
day2_2	0.027241	0.368192	0.493945
day2_3	0.031622	0.660879	0.546143
day2_4	0.203515	0.264171	0.297724
day2_5	0.189626	0.141543	0.159193
day2_6	0.104632	0.945191	0.963970
day2_7	0.041636	0.753362	0.897543
day2_8	0.039558	0.630168	0.837108
day2_9	0.051701	0.506006	0.374129
day2_10	0.043236	0.934157	0.988745
day2_11	0.125204	0.127061	0.074626
day2_12	0.002189	0.137949	0.165893
day2_13	0.165794	0.077676	0.102482
day2_14	0.332757	0.323087	0.056105
day2_15	0.194877	0.150283	0.244275
day2_16	0.395461	0.215757	0.200463
day2_17	0.306762	0.159721	0.501032
day2_18	0.061841	0.229706	0.408398
day2_19	0.026642	0.205501	0.298637
day2_20	0.031087	0.537367	0.818711
day2_21	0.061682	0.657445	0.380029
day2_22	0.068262	0.831112	0.543999
day2_23	0.037207	0.118302	0.220595
day3_0	0.048063	0.068837	0.230035
day3_1	0.030572	0.455683	0.789036
day3_2	0.018679	0.793748	0.956666
day3_3	0.013594	0.876933	0.946143
day3_4	0.115443	0.111029	0.145162
day3_5	0.395273	0.120822	0.618553
day3_6	0.475062	0.622409	0.962025
day3_7	0.164628	0.108740	0.202119

Table S2. PIR *P*-values calculated using ANOVA for each hourly bin in a linear model regressed against genotype, sex and genotype x sex interaction during the 12h light:12h dark related to Figure 2B.

PIR bins (day_hour)	*Genotype*	*Sex*	*Genotype x Sex*
day0_15	0.291880	0.000029	0.303824
day0_16	0.512743	0.710378	0.142128
day0_17	0.525148	0.312818	0.985833
day0_18	0.884841	0.484289	0.448198
day0_19	0.058697	0.063383	0.086968
day0_20	0.009481	0.019218	0.987767
day0_21	0.014714	0.810834	0.946675
day0_22	0.040755	0.907808	0.694890
day0_23	0.021338	0.954021	0.883057
day1_00	0.135730	0.146984	0.566090
day1_01	0.230322	0.650645	0.180709
day1_02	0.176603	0.319475	0.105586
day1_03	0.866130	0.182479	0.315435
day1_04	0.662698	0.040560	0.803224
day1_05	0.524620	0.418571	0.671911
day1_06	0.221139	0.242822	0.519588
day1_07	0.449274	0.433212	0.412749
day1_08	0.002604	0.096978	0.519744
day1_09	0.764321	0.413864	0.498157
day1_10	0.376280	0.000456	0.805293
day1_11	0.425307	0.208307	0.607628
day1_12	0.526479	0.593471	0.888795
day1_13	0.817096	0.450713	0.396564
day1_14	0.200992	0.032108	0.480506
day1_15	0.114047	0.945923	0.488306
day1_16	0.011507	0.611673	0.785221
day1_17	0.245017	0.828269	0.889075
day1_18	0.075592	0.712292	0.922986
day1_19	0.030646	0.024158	0.765968
day1_20	0.111679	0.897767	0.358449
day1_21	0.426920	0.187153	0.935467
day1_22	0.003841	0.927888	0.301239
day1_23	0.054432	0.578211	0.640915
day2_00	0.006650	0.534479	0.367962
day2_01	0.100753	0.897363	0.765709
day2_02	0.072179	0.892847	0.319260
day2_03	0.096114	0.302287	0.837084
day2_04	0.006234	0.371108	0.172005
day2_05	0.116855	0.111464	0.859674
day2_06	0.395541	0.530946	0.781514
day2_07	0.024390	0.720508	0.307632
day2_08	0.104652	0.426398	0.908680
day2_09	0.133190	0.006874	0.064053
day2_10	0.109180	0.066921	0.076277
day2_11	0.347115	0.151370	0.868355
day2_12	0.873967	0.029785	0.958172
day2_13	0.431739	0.149309	0.613961
day2_14	0.543356	0.015357	0.705892
day2_15	0.730810	0.341697	0.840381
day2_16	0.903769	0.862689	0.694037
day2_17	0.270176	0.036532	0.718709
day2_18	0.680221	0.113754	0.632620
day2_19	0.298785	0.205563	0.403227
day2_20	0.312699	0.483540	0.116974
day2_21	0.314452	0.074929	0.314630
day2_22	0.003515	0.002864	0.173981
day2_23	0.008269	0.422587	0.870283
day3_00	0.017414	0.459026	0.618970
day3_01	0.001063	0.460414	0.402747
day3_02	0.003365	0.524608	0.842532
day3_03	0.000467	0.100033	0.300934
day3_04	0.017854	0.047656	0.082018
day3_05	0.029069	0.185347	0.670308
day3_06	0.202286	0.547957	0.684463
day3_07	0.003029	0.550813	0.499447
day3_08	0.109763	0.864918	0.445532
day3_09	0.889790	0.001511	0.457180
day3_10	0.905018	0.179225	0.136523
day3_11	0.220457	0.038995	0.771361
day3_12	0.395171	0.455324	0.078963
day3_13	0.445190	0.032179	0.146039
day3_14	0.905106	0.060090	0.850352

day3_15	0.536419	0.081144	0.417934
day3_16	0.720492	0.620831	0.330140
day3_17	0.089888	0.482873	0.319513
day3_18	0.048731	0.637236	0.754456
day3_19	0.333947	0.390547	0.112800
day3_20	0.058497	0.590168	0.105656
day3_21	0.090988	0.033854	0.048983
day3_22	0.035241	0.014377	0.859571
day3_23	0.014622	0.465968	0.911903
day4_00	0.004553	0.096302	0.951124
day4_01	0.328172	0.736693	0.791693
day4_02	0.046969	0.493347	0.918867
day4_03	0.032912	0.568632	0.748041
day4_04	0.022244	0.038817	0.909334
day4_05	0.062422	0.906407	0.820557
day4_06	0.083224	0.553694	0.246131
day4_07	0.020218	0.735867	0.361011
day4_08	0.238593	0.924645	0.175272
day4_09	0.425779	0.000162	0.385928
day4_10	0.392660	0.574346	0.324686
day4_11	0.690229	0.749358	0.164855
day4_12	0.203677	0.022257	0.318552
day4_13	0.921054	0.480539	0.134591
day4_14	0.983071	0.271799	0.973401
day4_15	0.220437	0.204229	0.091229
day4_16	0.611556	0.296986	0.027604
day4_17	0.490122	0.752072	0.556878
day4_18	0.942570	0.532125	0.664478
day4_19	0.003574	0.018762	0.638312
day4_20	0.004473	0.458470	0.122036
day4_21	0.165871	0.233020	0.117489
day4_22	0.011176	0.853808	0.186556
day4_23	0.584579	0.070383	0.465681
day5_00	0.025516	0.290987	0.335228
day5_01	0.007287	0.555136	0.590615
day5_02	0.077679	0.292961	0.452928
day5_03	0.096257	0.754609	0.654831
day5_04	0.007884	0.373363	0.603898
day5_05	0.153687	0.946162	0.405889
day5_06	0.099872	0.021801	0.347171

Table S3. PIR *P*-values calculated using ANOVA for each hourly bin regressed against genotype, sex and genotype x sex interaction during constant darkness related to Figure 2C.

PIR bins (hour_day)	***Genotype***	***Sex***	***Genotype x Sex***
day5_07	0.060368	0.597579	0.204465
day5_08	0.101574	0.059578	0.221237
day5_09	0.047356	0.858431	0.806756
day5_10	0.236781	0.777324	0.697936
day5_11	0.564161	0.437503	0.749648
day5_12	0.316009	0.443059	0.290154
day5_13	0.088845	0.188380	0.308589
day5_14	0.326988	0.052546	0.243831
day5_15	0.883190	0.949368	0.522287
day5_16	0.289065	0.978510	0.030803
day5_17	0.835998	0.153555	0.990942
day5_18	0.701705	0.025959	0.031765
day5_19	0.124708	0.122235	0.536458
day5_20	0.028553	0.574658	0.070140
day5_21	0.153739	0.019787	0.837345
day5_22	0.003571	0.001740	0.104133
day5_23	0.000524	0.365331	0.652849
day6_00	0.000112	0.117780	0.032247
day6_01	0.001714	0.000054	0.005705
day6_02	0.117292	0.739229	0.824558
day6_03	0.010330	0.831585	0.587258
day6_04	0.014268	0.118014	0.251773
day6_05	0.070766	0.906061	0.714054
day6_06	0.194494	0.907383	0.929910
day6_07	0.501487	0.652166	0.016954
day6_08	0.135913	0.239154	0.119667
day6_09	0.090481	0.000360	0.821690
day6_10	0.231177	0.601734	0.617802
day6_11	0.404191	0.655037	0.454109
day6_12	0.943860	0.241520	0.126762
day6_13	0.606614	0.503709	0.884464
day6_14	0.880710	0.030563	0.639208
day6_15	0.222757	0.688664	0.417498
day6_16	0.356801	0.437825	0.604997
day6_17	0.420634	0.529328	0.731821
day6_18	0.654145	0.196745	0.031453
day6_19	0.034559	0.165153	0.142609
day6_20	0.019105	0.008312	0.445610
day6_21	0.021527	0.845034	0.524368
day6_22	0.013001	0.866063	0.862554
day6_23	0.107999	0.923726	0.295248
day7_00	0.009030	0.078035	0.328741
day7_01	0.000077	0.490328	0.255684
day7_02	0.010961	0.159248	0.342390
day7_03	0.000731	0.268607	0.161003
day7_04	0.044469	0.111742	0.845200
day7_05	0.383946	0.053271	0.263422
day7_06	0.025985	0.643479	0.828048
day7_07	0.225687	0.462577	0.043634
day7_08	0.124211	0.952356	0.963073
day7_09	0.109061	0.017734	0.094926
day7_10	0.292774	0.641854	0.063118
day7_11	0.155178	0.532628	0.510668
day7_12	0.699816	0.226797	0.236276
day7_13	0.850507	0.298848	0.298252
day7_14	0.082575	0.003188	0.047622
day7_15	0.841745	0.068409	0.031514
day7_16	0.587504	0.711772	0.433957
day7_17	0.758273	0.671627	0.429526
day7_18	0.541958	0.018065	0.787111
day7_19	0.950760	0.645265	0.167494
day7_20	0.849264	0.290433	0.211929
day7_21	0.442009	0.696390	0.007579
day7_22	0.054283	0.605004	0.488194
day7_23	0.125207	0.702605	0.870615
day8_00	0.003107	0.395374	0.294435
day8_01	0.016094	0.196102	0.482472
day8_02	0.037919	0.560020	0.810358
day8_03	0.014211	0.112814	0.589104
day8_04	0.072450	0.767598	0.342951
day8_05	0.671024	0.847509	0.897790
day8_06	0.108637	0.830903	0.540034
day8_07	0.016752	0.696709	0.762819
day8_08	0.105055	0.615572	0.035659
day8_09	0.121253	0.024249	0.229410

day8_10	0.211844	0.317289	0.213587
day8_11	0.966426	0.794671	0.971575
day8_12	0.107079	0.494541	0.730813
day8_13	0.497849	0.656609	0.956665
day8_14	0.686591	0.179185	0.867316
day8_15	0.714330	0.664855	0.043522
day8_16	0.579696	0.993929	0.420774
day8_17	0.617464	0.010075	0.804303
day8_18	0.619752	0.000005	0.722723
day8_19	0.419982	0.739840	0.503844
day8_20	0.884471	0.832157	0.442267
day8_21	0.517333	0.007486	0.676753
day8_22	0.779348	0.380591	0.195487
day8_23	0.143411	0.418226	0.996057
day9_00	0.108798	0.492348	0.332051
day9_01	0.616046	0.585246	0.108315
day9_02	0.066546	0.898774	0.722380
day9_03	0.069195	0.218174	0.765576
day9_04	0.487140	0.407551	0.199815
day9_05	0.155729	0.731252	0.456514
day9_06	0.160760	0.920384	0.501933
day9_07	0.042200	0.264585	0.139975
day9_08	0.371057	0.704579	0.029742
day9_09	0.217282	0.018876	0.551838
day9_10	0.137954	0.358582	0.147917
day9_11	0.206859	0.663234	0.387786
day9_12	0.259404	0.199788	0.625155
day9_13	0.329820	0.477264	0.156106
day9_14	0.238293	0.350624	0.384795
day9_15	0.906912	0.659397	0.258068
day9_16	0.339625	0.711053	0.352482
day9_17	0.092460	0.000025	0.245141
day9_18	0.857922	0.001548	0.435537
day9_19	0.030056	0.786199	0.901571
day9_20	0.381024	0.333527	0.284450
day9_21	0.079916	0.539662	0.411005
day9_22	0.087639	0.858871	0.580344
day9_23	0.117621	0.771041	0.546569
day10_00	0.012322	0.134721	0.117203
day10_01	0.002492	0.188574	0.723409
day10_02	0.167818	0.750351	0.615142
day10_03	0.002866	0.894824	0.899902
day10_04	0.086562	0.959689	0.416316
day10_05	0.280210	0.978537	0.523247
day10_06	0.162915	0.281983	0.435176
day10_07	0.129417	0.691790	0.014401
day10_08	0.192973	0.101039	0.172561
day10_09	0.127198	0.195444	0.623581
day10_10	0.622877	0.056891	0.520679
day10_11	0.716966	0.236248	0.260916
day10_12	0.621635	0.572553	0.605829
day10_13	0.872829	0.891615	0.498539
day10_14	0.186656	0.176739	0.200653
day10_15	0.591725	0.848718	0.613709
day10_16	0.524072	0.161103	0.752373
day10_17	0.419769	0.000029	0.198485
day10_18	0.536222	0.194083	0.887448
day10_19	0.671244	0.493889	0.937766
day10_20	0.903129	0.250692	0.694931
day10_21	0.032074	0.066552	0.518767
day10_22	0.945139	0.813424	0.261076
day10_23	0.055010	0.992926	0.311073
day11_00	0.021756	0.042239	0.162104
day11_01	0.001383	0.935844	0.533331
day11_02	0.007931	0.668253	0.512900
day11_03	0.008404	0.083359	0.133094
day11_04	0.082972	0.867729	0.343538
day11_05	0.020938	0.345389	0.571100
day11_06	0.780274	0.036711	0.070464
day11_07	0.130105	0.436587	0.568180
day11_08	0.210090	0.303218	0.237435
day11_09	0.222223	0.000009	0.441910
day11_10	0.269966	0.136228	0.187158
day11_11	0.264647	0.503667	0.975025
day11_12	0.661969	0.032471	0.479261
day11_13	0.486145	0.605235	0.143752
day11_14	0.114833	0.994000	0.437108
day11_15	0.598363	0.901766	0.251572
day11_16	0.725163	0.049048	0.326407
day11_17	0.700679	0.000001	0.112328

day11_18	0.232155	0.700077	0.562488
day11_19	0.566992	0.820033	0.199272
day11_20	0.797899	0.253745	0.789118
day11_21	0.109189	0.682175	0.592906
day11_22	0.156168	0.736541	0.886372
day11_23	0.000976	0.912903	0.307949
day12_00	0.069105	0.313949	0.546641
day12_01	0.044165	0.382820	0.906195
day12_02	0.001861	0.583214	0.878985
day12_03	0.073892	0.866388	0.405006
day12_04	0.218806	0.939273	0.222915
day12_05	0.130094	0.581807	0.707886
day12_06	0.111302	0.929478	0.611245
day12_07	0.045410	0.105317	0.200515
day12_08	0.122825	0.983791	0.741322
day12_09	0.397702	0.256077	0.148514
day12_10	0.867423	0.309697	0.262487
day12_11	0.068617	0.874408	0.033047
day12_12	0.586489	0.835409	0.250725
day12_13	0.967919	0.406948	0.521375
day12_14	0.857231	0.100848	0.751043
day12_15	0.562728	0.113025	0.741484
day12_16	0.093173	0.000039	0.118359
day12_17	0.764918	0.000002	0.628437
day12_18	0.723709	0.814575	0.685911
day12_19	0.982715	0.283424	0.554505
day12_20	0.893365	0.434569	0.105309
day12_21	0.489102	0.901725	0.694528
day12_22	0.108674	0.673207	0.346905
day12_23	0.130089	0.034723	0.228227
day13_00	0.115594	0.109388	0.461757
day13_01	0.010964	0.815896	0.894336
day13_02	0.086617	0.576458	0.603384
day13_03	0.104553	0.731597	0.931195
day13_04	0.271599	0.258746	0.528625
day13_05	0.010516	0.460879	0.322614
day13_06	0.101242	0.483849	0.369449
day13_07	0.552542	0.180038	0.327434
day13_08	0.119371	0.039784	0.284977
day13_09	0.003189	0.000000	0.708067
day13_10	0.375375	0.909848	0.646135
day13_11	0.289441	0.447459	0.182626
day13_12	0.400748	0.427291	0.259500
day13_13	0.812789	0.741029	0.883106
day13_14	0.992376	0.897036	0.733583
day13_15	0.583763	0.430563	0.326614
day13_16	0.568928	0.000001	0.096398
day13_17	0.630713	0.000102	0.896165
day13_18	0.655761	0.468864	0.616689
day13_19	0.311653	0.658090	0.372470
day13_20	0.059642	0.717355	0.856449
day13_21	0.283440	0.905344	0.641914
day13_22	0.196498	0.283180	0.119575
day13_23	0.103419	0.231385	0.521710
day14_00	0.071096	0.013820	0.734451
day14_01	0.018236	0.821168	0.876072
day14_02	0.113267	0.272741	0.411918
day14_03	0.154743	0.737276	0.766851
day14_04	0.260560	0.159027	0.619530
day14_05	0.027626	0.534578	0.745646
day14_06	0.184226	0.849436	0.859952
day14_07	0.096596	0.391875	0.291986
day14_08	0.062538	0.243977	0.262998
day14_09	0.596326	0.587124	0.358685
day14_10	0.510093	0.553055	0.120131
day14_11	0.212955	0.220451	0.358073

Table S4. Adjusted *P*-values for Tukey post-hoc multiple comparisons ANOVA for each genotype against measures of distance, duration and velocity in open field per each 5-minute bin related to Figure 2D-E and Figure S1 A-C.

Open field measure and bin	$Whrn^{+/+}$ vs $Whrn^{+/hb}$	$Whrn^{+/+}$ vs $Whrn^{hb/hb}$	$Whrn^{+/+}$ vs $Whrn^{hb/wi}$	$Whrn^{+/+}$ vs $Whrn^{hb/hb;}$ BAC279+/-	$Whrn^{+/+}$ vs $Whrn^{hb/wi;}$ BAC279+/-	$Whrn^{+/hb}$ vs $Whrn^{hb/hb}$	$Whrn^{+/hb}$ vs $Whrn^{hb/wi}$	$Whrn^{+/hb}$ vs $Whrn^{hb/hb;}$ BAC279+/-	$Whrn^{+/hb}$ vs $Whrn^{hb/wi}$;BAC279+/-	$Whrn^{hb/hb}$ vs $Whrn^{hb/wi}$	$Whrn^{hb/hb}$ vs $Whrn^{hb/hb;}$ BAC279+/-	$Whrn^{hb/hb}$ vs $Whrn^{hb/wi;}$ BAC279+/-	$Whrn^{hb/wi}$ vs $Whrn^{hb/hb;}$ BAC279+/-	$Whrn^{hb/wi}$ vs $Whrn^{hb/wi}$;BAC279+/-	$Whrn^{hb/hb;}$ BAC279+/- vs $Whrn^{hb/wi;}$ BAC279+/-
Centre distance 0-5	1.000000	0.262632	0.989167	0.995791	0.999993	0.446980	0.997796	0.994117	0.999957	0.580729	0.035680	0.316691	0.727563	0.978228	0.999924
Centre distance 5-10	0.999995	0.881044	0.966388	0.997986	1.000000	0.846579	0.943394	0.999954	1.000000	0.999657	0.484515	0.924361	0.653805	0.980196	0.999595
Centre distance 10-15	0.999998	0.479939	0.430465	0.999271	0.999962	0.492399	0.457344	0.999984	0.999670	1.000000	0.154004	0.822239	0.107078	0.814076	0.993333
Centre distance 15-20	1.000000	0.414233	0.308057	1.000000	1.000000	0.560022	0.471383	1.000000	1.000000	1.000000	0.288598	0.630339	0.181980	0.553349	1.000000
Peripheral distance 0-5	0.973358	0.644175	0.130158	0.531654	0.975047	0.203688	0.021179	0.993151	0.678992	0.978939	0.006149	0.998295	0.000049	0.868444	0.174413
Peripheral distance 5-10	0.999926	0.222650	0.002174	0.999992	0.969076	0.186050	0.003104	0.999999	0.918879	0.762052	0.087051	0.926961	0.000186	0.221151	0.910251
Peripheral distance 10-15	0.999914	0.004508	0.000009	0.998729	0.953185	0.039234	0.000446	1.000000	0.995051	0.852910	0.008916	0.288356	0.000010	0.014602	0.995762
Peripheral distance 15-20	1.000000	0.027120	0.000023	0.999013	0.942446	0.084452	0.000381	0.999907	0.975374	0.621256	0.051568	0.617110	0.000027	0.029284	0.992703
Total distance 0-5	0.993386	0.351748	0.207532	0.594042	0.990287	0.133195	0.068791	0.981903	0.857942	1.000000	0.001515	0.935015	0.000206	0.878708	0.277717
Total distance 5-10	0.999747	0.176203	0.013880	0.999832	0.995377	0.125907	0.012260	1.000000	0.966794	0.986284	0.042585	0.754498	0.001045	0.298992	0.957877
Total distance 10-15	0.999997	0.008161	0.000114	0.999978	0.979319	0.041832	0.001890	1.000000	0.995967	0.974399	0.007028	0.286151	0.000051	0.039429	0.993319
Total distance 15-20	1.000000	0.043245	0.000695	0.999929	0.993932	0.116942	0.005471	0.999997	0.998141	0.947205	0.050813	0.457815	0.000504	0.066237	0.999356
Centre duration 0-5	1.000000	0.357035	0.999883	0.990652	0.999998	0.557484	0.999997	0.987673	0.999980	0.428467	0.045547	0.434767	0.898417	0.999289	0.999391
Centre duration 5-10	0.997031	0.676046	0.999929	0.982916	1.000000	0.978427	0.999811	0.823050	0.999223	0.763141	0.139041	0.839921	0.870746	0.999995	0.988374
Centre duration 10-15	0.999906	0.300295	0.814846	0.996484	0.998162	0.242061	0.700073	0.999994	0.985819	0.946197	0.047265	0.828048	0.306231	0.997335	0.930694
Centre duration 15-20	0.999986	0.002893	0.558851	1.000000	0.999991	0.021814	0.831658	0.999992	0.999639	0.175939	0.000994	0.008723	0.467824	0.590732	0.999974
Peripheral duration 0-5	0.990764	0.459455	0.999998	1.000000	1.000000	0.950679	0.995695	0.994168	0.998024	0.439852	0.432929	0.705491	1.000000	1.000000	1.000000
Peripheral duration 5-10	0.999993	0.485656	1.000000	0.996971	0.999378	0.743341	0.999999	0.988910	0.996724	0.404330	0.115477	0.365758	0.989179	0.998096	1.000000
Peripheral duration 10-15	1.000000	0.223647	0.952860	1.000000	0.999604	0.273356	0.948089	1.000000	0.998949	0.691146	0.131286	0.674552	0.909029	0.999666	0.999084
Peripheral duration 15-20	1.000000	0.084551	0.962902	1.000000	0.916455	0.158187	0.978820	1.000000	0.941028	0.349614	0.034815	0.009141	0.910645	0.406089	0.920262
Total velocity 0-5	0.993413	0.351869	0.207577	0.594051	0.990326	0.133414	0.068909	0.981842	0.858382	1.000000	0.001516	0.934908	0.000206	0.878500	0.278024
Total velocity 5-10	0.999747	0.176214	0.013830	0.999832	0.995378	0.125958	0.012226	1.000000	0.966818	0.986173	0.042590	0.754509	0.001040	0.298471	0.957879
Total velocity 10-15	0.999997	0.008148	0.000114	0.999978	0.979217	0.041781	0.001889	1.000000	0.995940	0.974459	0.007026	0.286366	0.000051	0.039515	0.993293
Total velocity 15-200	1.000000	0.043178	0.000694	0.999928	0.993910	0.116791	0.005460	0.999997	0.998132	0.947210	0.050817	0.457798	0.000504	0.066236	0.999356

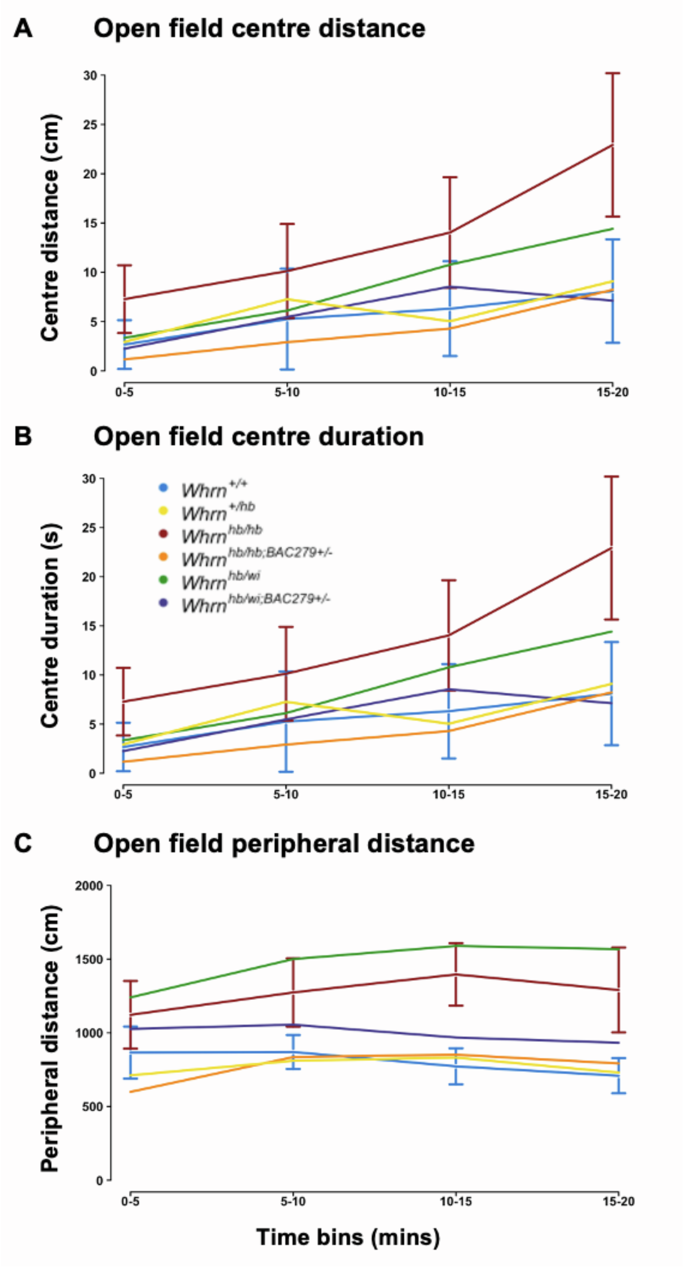

Figure S1. Open field centre and periphery activity related to Figure 2D-E. (A-B) Distance and duration in the open field showed few differences across genotypes for the first 3 bins. The final bin revealed significant (*P*<0.05) differences between *Whrn*$^{+/+}$ (n=20) and *Whrn*$^{hb/hb}$ (n=22) reflecting hyperactivity in mutants rather than specific anxiety-related behaviours. Data are represented as mean +/- confidence interval width (95%) for *Whrn*$^{+/+}$ and *Whrn*$^{hb/hb}$. (C) Peripheral distance activity reveals significant (*P*<0.05) hyperactivity across the last 3 bins in *Whrn*$^{hb/hb}$ compared with *Whrn*$^{+/+}$.

Table S5. Adjusted *P*-values for Tukey post-hoc multiple comparisons ANOVA for each genotype against ABR frequency related to Figure 3A.

ANOVA comparison	*Frequency*	*Genotype difference*	*Lower limit*	*Upper limit*	*P adjusted*
Whrn$^{+/+}$ vs *Whrn*$^{+/hb}$		1.670000	-6.753000	10.086000	0.995890
Whrn$^{+/+}$ vs *Whrn*$^{hb/hb}$		-17.500000	-25.920000	-9.080000	0.000003
Whrn$^{+/+}$ vs *Whrn*$^{hb/hb;BAC279+/-}$		-16.670000	-25.086000	-8.247000	0.000007
Whrn$^{+/+}$ vs *Whrn*$^{hb/wi}$		-22.500000	-30.920000	-14.080000	0.000000
Whrn$^{+/+}$ vs *Whrn*$^{hb/wi;BAC279+/-}$		-22.500000	-30.920000	-14.080000	0.000000
Whrn$^{+/+}$ vs *Whrn*$^{wi/wi}$		-57.780000	-65.464000	-50.092000	0.000000
Whrn$^{+/hb}$ vs *Whrn*$^{hb/hb}$		19.170000	10.747000	27.586000	0.000000
Whrn$^{+/hb}$ vs *Whrn*$^{hb/hb;BAC279+/-}$		-18.330000	-26.753000	-9.914000	0.000001
Whrn$^{+/hb}$ vs *Whrn*$^{hb/wi}$		-24.170000	-32.586000	-15.747000	0.000000
Whrn$^{+/hb}$ vs *Whrn*$^{hb/wi;BAC279+/-}$		-24.170000	-32.586000	-15.747000	0.000000
Whrn$^{+/hb}$ vs *Whrn*$^{wi/wi}$	8khz	59.440000	51.758000	67.130000	0.000000
Whrn$^{hb/hb}$ vs *Whrn*$^{hb/hb;BAC279+/-}$		0.830000	-7.586000	9.253000	0.999921
Whrn$^{hb/hb}$ vs *Whrn*$^{hb/wi}$		-5.000000	-13.420000	3.420000	0.525689
Whrn$^{hb/hb}$ vs *Whrn*$^{hb/wi;BAC279+/-}$		-5.000000	-13.420000	3.420000	0.525689
Whrn$^{hb/hb}$ vs *Whrn*$^{wi/wi}$		40.280000	32.592000	47.964000	0.000000
Whrn$^{hb/hb;BAC279+/-}$ vs *Whrn*$^{hb/wi}$		5.830000	-2.586000	14.253000	0.342650
Whrn$^{hb/hb;BAC279+/-}$ vs *Whrn*$^{hb/wi;BAC279+/-}$		5.830000	-2.586000	14.253000	0.342650
Whrn$^{hb/hb;BAC279+/-}$ vs *Whrn*$^{wi/wi}$		41.110000	33.425000	48.797000	0.000000
Whrn$^{hb/wi}$ vs *Whrn*$^{hb/wi;BAC279+/-}$		0.000000	-8.420000	8.420000	1.000000
Whrn$^{hb/wi}$ vs *Whrn*$^{wi/wi}$		35.280000	27.592000	42.964000	0.000000
Whrn$^{hb/hb;BAC279+/-}$ vs *Whrn*$^{wi/wi}$		35.280000	27.592000	42.964000	0.000000
Whrn$^{+/+}$ vs *Whrn*$^{+/hb}$		0.830000	-7.725000	9.392000	0.999928
Whrn$^{+/+}$ vs *Whrn*$^{hb/hb}$		-15.830000	-24.392000	-7.275000	0.000024
Whrn$^{+/+}$ vs *Whrn*$^{hb/hb;BAC279+/-}$		-16.670000	-25.225000	-8.108000	0.000009
Whrn$^{+/+}$ vs *Whrn*$^{hb/wi}$		-27.500000	-36.058000	-18.942000	0.000000
Whrn$^{+/+}$ vs *Whrn*$^{hb/wi;BAC279+/-}$		-25.000000	-33.558000	-16.442000	0.000000
Whrn$^{+/+}$ vs *Whrn*$^{wi/wi}$		-67.500000	-75.313000	-59.687000	0.000000
Whrn$^{+/hb}$ vs *Whrn*$^{hb/hb}$		16.670000	8.108000	25.225000	0.000009
Whrn$^{+/hb}$ vs *Whrn*$^{hb/hb;BAC279+/-}$		-17.500000	-26.058000	-8.942000	0.000004
Whrn$^{+/hb}$ vs *Whrn*$^{hb/wi}$		-28.330000	-36.892000	-19.775000	0.000000
Whrn$^{+/hb}$ vs *Whrn*$^{hb/wi;BAC279+/-}$		-25.830000	-34.392000	-17.275000	0.000000
Whrn$^{+/hb}$ vs *Whrn*$^{wi/wi}$	16khz	68.330000	60.521000	76.146000	0.000000
Whrn$^{hb/hb}$ vs *Whrn*$^{hb/hb;BAC279+/-}$		-0.830000	-9.392000	7.725000	0.999928
Whrn$^{hb/hb}$ vs *Whrn*$^{hb/wi}$		-11.670000	-20.225000	-3.108000	0.002417
Whrn$^{hb/hb}$ vs *Whrn*$^{hb/wi;BAC279+/-}$		-9.170000	-17.725000	-0.608000	0.029023
Whrn$^{hb/hb}$ vs *Whrn*$^{wi/wi}$		51.670000	43.854000	59.479000	0.000000
Whrn$^{hb/hb;BAC279+/-}$ vs *Whrn*$^{hb/wi}$		10.830000	2.275000	19.392000	0.005741
Whrn$^{hb/hb;BAC279+/-}$ vs *Whrn*$^{hb/wi;BAC279+/-}$		8.330000	-0.225000	16.892000	0.060678
Whrn$^{hb/hb;BAC279+/-}$ vs *Whrn*$^{wi/wi}$		50.830000	43.021000	58.646000	0.000000
Whrn$^{hb/wi}$ vs *Whrn*$^{hb/wi;BAC279+/-}$		2.500000	-6.058000	11.058000	0.968882
Whrn$^{hb/wi}$ vs *Whrn*$^{wi/wi}$		40.000000	32.187000	47.813000	0.000000
Whrn$^{hb/hb;BAC279+/-}$ vs *Whrn*$^{wi/wi}$		42.500000	34.687000	50.313000	0.000000
Whrn$^{+/+}$ vs *Whrn*$^{+/hb}$		-0.833000	-10.407000	8.740000	0.999963
Whrn$^{+/+}$ vs *Whrn*$^{hb/hb}$		-27.500000	-37.073000	-17.927000	0.000000
Whrn$^{+/+}$ vs *Whrn*$^{hb/hb;BAC279+/-}$		-25.000000	-34.573000	-15.427000	0.000000
Whrn$^{+/+}$ vs *Whrn*$^{hb/wi}$		-37.500000	-47.073000	-27.927000	0.000000
Whrn$^{+/+}$ vs *Whrn*$^{hb/wi;BAC279+/-}$		-33.330000	-42.907000	-23.760000	0.000000
Whrn$^{+/+}$ vs *Whrn*$^{wi/wi}$		-56.670000	-65.406000	-47.927000	0.000000
Whrn$^{+/hb}$ vs *Whrn*$^{hb/hb}$		26.670000	17.093000	36.240000	0.000000
Whrn$^{+/hb}$ vs *Whrn*$^{hb/hb;BAC279+/-}$		-24.170000	-33.740000	-14.593000	0.000000
Whrn$^{+/hb}$ vs *Whrn*$^{hb/wi}$		-36.670000	-46.240000	-27.093000	0.000000
Whrn$^{+/hb}$ vs *Whrn*$^{hb/wi;BAC279+/-}$		-32.500000	-42.073000	-22.927000	0.000000
Whrn$^{+/hb}$ vs *Whrn*$^{wi/wi}$	32khz	55.830000	47.094000	64.573000	0.000000
Whrn$^{hb/hb}$ vs *Whrn*$^{hb/hb;BAC279+/-}$		2.500000	-7.073000	12.073000	0.982204
Whrn$^{hb/hb}$ vs *Whrn*$^{hb/wi}$		-10.000000	-19.573000	-0.427000	0.035669
Whrn$^{hb/hb}$ vs *Whrn*$^{hb/wi;BAC279+/-}$		-5.830000	-15.407000	3.740000	0.495266
Whrn$^{hb/hb}$ vs *Whrn*$^{wi/wi}$		29.170000	20.427000	37.906000	0.000000
Whrn$^{hb/hb;BAC279+/-}$ vs *Whrn*$^{hb/wi}$		12.500000	2.927000	22.073000	0.004042
Whrn$^{hb/hb;BAC279+/-}$ vs *Whrn*$^{hb/wi;BAC279+/-}$		8.330000	-1.240000	17.907000	0.123781
Whrn$^{hb/hb;BAC279+/-}$ vs *Whrn*$^{wi/wi}$		31.670000	22.927000	40.406000	0.000000
Whrn$^{hb/wi}$ vs *Whrn*$^{hb/wi;BAC279+/-}$		4.170000	-5.407000	13.740000	0.821766
Whrn$^{hb/wi}$ vs *Whrn*$^{wi/wi}$		19.170000	10.427000	27.906000	0.000001
Whrn$^{hb/hb;BAC279+/-}$ vs *Whrn*$^{wi/wi}$		23.330000	14.594000	32.073000	0.000000
Whrn$^{+/+}$ vs *Whrn*$^{+/hb}$		0.000000	-8.135000	8.135000	1.000000
Whrn$^{+/+}$ vs *Whrn*$^{hb/hb}$		-24.170000	-32.301000	-16.032000	0.000000
Whrn$^{+/+}$ vs *Whrn*$^{hb/hb;BAC279+/-}$		-10.000000	-18.135000	-1.865000	0.007872
Whrn$^{+/+}$ vs *Whrn*$^{hb/wi}$		-29.170000	-37.301000	-21.032000	0.000000
Whrn$^{+/+}$ vs *Whrn*$^{hb/wi;BAC279+/-}$		-32.500000	-40.635000	-24.365000	0.000000
Whrn$^{+/+}$ vs *Whrn*$^{wi/wi}$		-60.000000	-67.426000	-52.574000	0.000000
Whrn$^{+/hb}$ vs *Whrn*$^{hb/hb}$		24.170000	16.032000	32.301000	0.000000
Whrn$^{+/hb}$ vs *Whrn*$^{hb/hb;BAC279+/-}$		-10.000000	-18.135000	-1.865000	0.007872
Whrn$^{+/hb}$ vs *Whrn*$^{hb/wi}$		-29.170000	-37.301000	-21.032000	0.000000
Whrn$^{+/hb}$ vs *Whrn*$^{hb/wi;BAC279+/-}$		-32.500000	-40.635000	-24.365000	0.000000
Whrn$^{+/hb}$ vs *Whrn*$^{wi/wi}$	click	60.000000	52.574000	67.426000	0.000000
Whrn$^{hb/hb}$ vs *Whrn*$^{hb/hb;BAC279+/-}$		14.170000	6.032000	22.301000	0.000069
Whrn$^{hb/hb}$ vs *Whrn*$^{hb/wi}$		-5.000000	-13.135000	3.135000	0.484891
Whrn$^{hb/hb}$ vs *Whrn*$^{hb/wi;BAC279+/-}$		-8.330000	-16.468000	-0.199000	0.041609
Whrn$^{hb/hb}$ vs *Whrn*$^{wi/wi}$		35.830000	28.408000	43.259000	0.000000
Whrn$^{hb/hb;BAC279+/-}$ vs *Whrn*$^{hb/wi}$		19.170000	11.032000	27.301000	0.000000
Whrn$^{hb/hb;BAC279+/-}$ vs *Whrn*$^{hb/wi;BAC279+/-}$		22.500000	14.365000	30.635000	0.000000
Whrn$^{hb/hb;BAC279+/-}$ vs *Whrn*$^{wi/wi}$		50.000000	42.574000	57.426000	0.000000
Whrn$^{hb/wi}$ vs *Whrn*$^{hb/wi;BAC279+/-}$		-3.330000	-11.468000	4.801000	0.858900
Whrn$^{hb/wi}$ vs *Whrn*$^{wi/wi}$		30.830000	23.408000	38.259000	0.000000
Whrn$^{hb/hb;BAC279+/-}$ vs *Whrn*$^{wi/wi}$		27.500000	20.074000	34.926000	0.000000

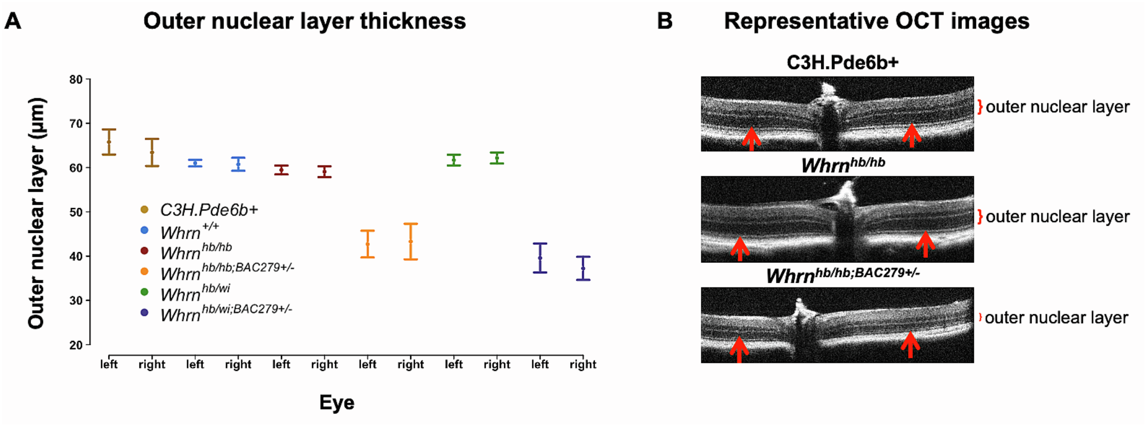

Figure S2. OCT measures of the outer nuclear layer. (A-B) Outer nuclear layer thickness measured across *Whrn* genotypes (C3H.Pde6b+, n= 6; *Whrn*$^{+/+}$, n=16; *Whrn*$^{hb/hb}$, n=16; *Whrn*$^{hb/hb;BAC279+/-}$, n=15; *Whrn*$^{hb/wi}$, n=16; *Whrn*$^{hb/hb}$, n=16; *Whrn*$^{hb/wi; BAC279+/-}$, n=9;) indicated a significantly (*P*<0.05) reduced outer nuclear layer thickness between BAC279 mutants and each of the genotypes potentially attributed to a genetic background effect. Data are represented as mean +/- confidence interval width (95%).

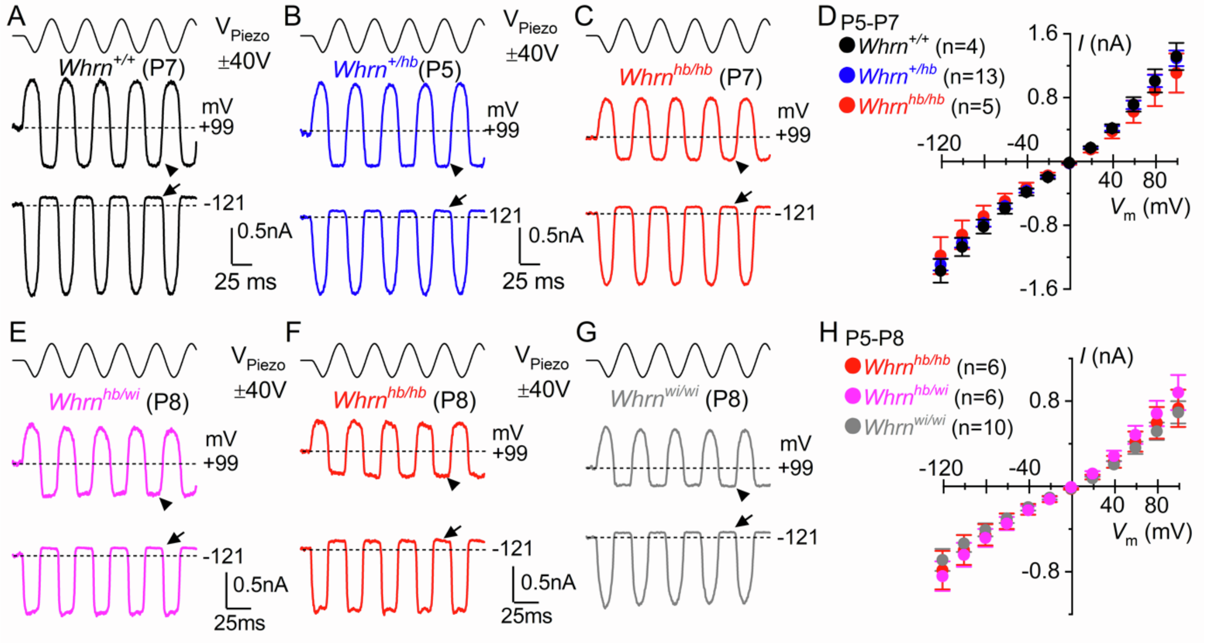

Figure S3. Mechanoelectrical transducer currents in apical OHCs related to Potential loss of Ca^{2+} sensitivity of the MET current. Saturating mechanoelectrical transducer (MET) currents recorded from *Whrn*$^{+/+}$ (A), *Whrn*$^{+/hb}$ (B) and *Whrn*$^{hb/hb}$ (C) apical-coil OHCs by applying sinusoidal force stimuli of 50 Hz to the hair bundles when held at -121 mV and +99 mV. The driver voltage (DV) signal of ± 40 V to the fluid jet is shown above the traces (positive deflections of the DV are excitatory). Between stimuli, the OHC holding potential was -81 mV. The arrows and arrowheads indicate the closure of the MET currents (resting MET current) elicited during inhibitory bundle displacements at hyperpolarised and depolarised membrane potentials, respectively. Dashed lines indicate the holding current at each membrane potential. (D) Average current-voltage curves (no MET currents activated) recorded from OHCs of *Whrn*$^{+/+}$ (n=4), *Whrn*$^{+/hb}$ (n=13), and *Whrn*$^{hb/hb}$ (n=5) at P5-P7, indicating the number of OHCs recorded in parentheses. Data represent mean +/- SEM. (E-G), Saturating MET currents from OHCs of *Whrn*$^{hb/wi}$, *Whrn*$^{hb/hb}$ and *Whrn*$^{wi/wi}$ mice. MET channel resting P_{open} was comparable across whirlin mutants at -121 mV (*Whrn*$^{hb/wi}$: 9.2 ± 1%, *Whrn*$^{hb/hb}$: 7.5 ± 1%; *Whrn*$^{wi/wi}$: 6.3 ± 1%, P = 0.2929), or, +99 mV (*Whrn*$^{hb/wi}$: 40.2 ± 4%; *Whrn*$^{hb/hb}$: 32.5 ± 6%; *Whrn*$^{wi/wi}$: 22.9 ± 5%, P = 0.0645). At positive P_{open} at +99 mV, all mutants are significantly different to *Whrn*$^{+/+}$ (58 ± 5%, n = 4), arrows and arrowheads as above. (H) Average current-voltage curves recorded from OHCs (P5-P8) of *Whrn*$^{hb/wi}$ (n = 6), *Whrn*$^{hb/hb}$ (n = 6) and *Whrn*$^{wi/wi}$ (n = 10), indicating number of OHCs recorded in parentheses. Data represent mean +/- SEM.

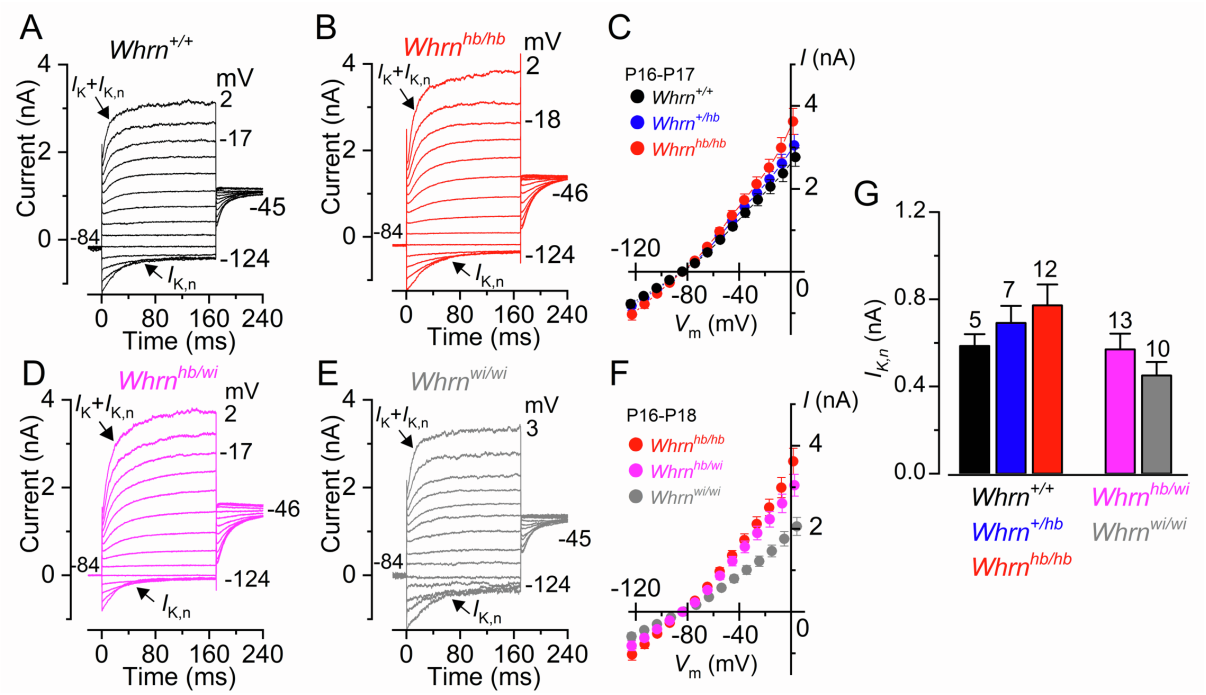

Figure S4. Current responses in mature OHCs. Currents recorded from mature *Whrn*$^{+/+}$ (A) and *Whrn*$^{hb/hb}$ (B) apical coil OHCs at P16, elicited by depolarising voltage steps (10 mV nominal increments) from -124 mV to more depolarised values from the holding potential of -84 mV. Note that the characteristic current of mature OHCs, $I_{K,n}$, was present in both genotypes. (C) Average peak current-voltage relationship for the total K^+ current recorded in *Whrn*$^{+/+}$ (n=5), *Whrn*$^{+/hb}$ (n=7) and *Whrn*$^{hb/hb}$ (n=12) P16-P17 OHCs. (D-E) Currents recorded from mature *Whrn*$^{hb/wi}$ and *Whrn*$^{wi/wi}$ apical coil OHCs at P18. (F) Average peak current-voltage relationship for the K^+ current recorded from *Whrn*$^{hb/hb}$ (n=12), *Whrn*$^{hb/wi}$ (n=13) and *Whrn*$^{wi/wi}$ (n=10) OHCs at P16-P18. (G) $I_{K,n}$, measured as the deactivating tail currents (difference between instantaneous and steady-state inward currents) for voltage steps from the holding potential to -124 mV. Data represent mean +/- SEM.

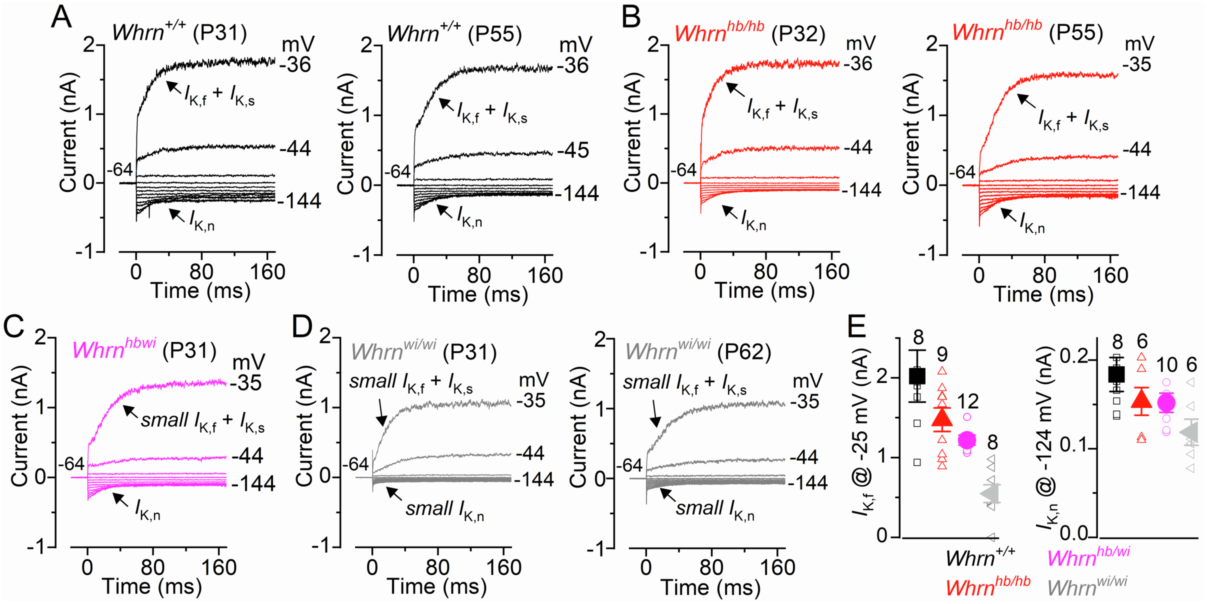

Figure S5. Current responses in mature IHCs related to Mutant IHCs lack maturation of electrophysiological traits. Currents recorded from mature apical-coil IHCs from *Whrn*$^{+/+}$ (A, P31 and P55), *Whrn*$^{hb/hb}$ (B, P32 and P55), *Whrn*$^{hb/wi}$ (C, P31) and *Whrn*$^{wi/wi}$ (D, P31 and P62), elicited by depolarising voltage steps (10 mV nominal increments) from -144 mV to more depolarised values from the holding potential of -64 mV. (E) Average size of the isolated $I_{K,f}$, measured at 1.0 ms after stimulus onset at a membrane potential of -25 mV (left panel), and $I_{K,n}$ (right panel). Note that numbers within the plots represent IHCs recorded. Tukey post-hoc significance in one-way ANOVA for $I_{K,f}$ showed: P<0.001 for *Whrn*$^{+/+}$ vs *Whrn*$^{wi/wi}$; P <0.01 for *Whrn*$^{hb/hb}$ vs *Whrn*$^{hb/wi}$; P <0.05 for *Whrn*$^{+/+}$ vs *Whrn*$^{hb/wi}$ and *Whrn*$^{hb/wi}$ vs *Whrn*$^{wi/wi}$; P >0.05 for *Whrn*$^{+/+}$ vs *Whrn*$^{hb/hb}$ and *Whrn*$^{hb/hb}$ vs *Whrn*$^{hb/wi}$. For $I_{K,n}$, *Whrn*$^{+/+}$ and *Whrn*$^{hb/wi}$ were significantly different (P <0.05); summary one-way ANOVA: P = 0.0581. Data represent mean +/- SEM.

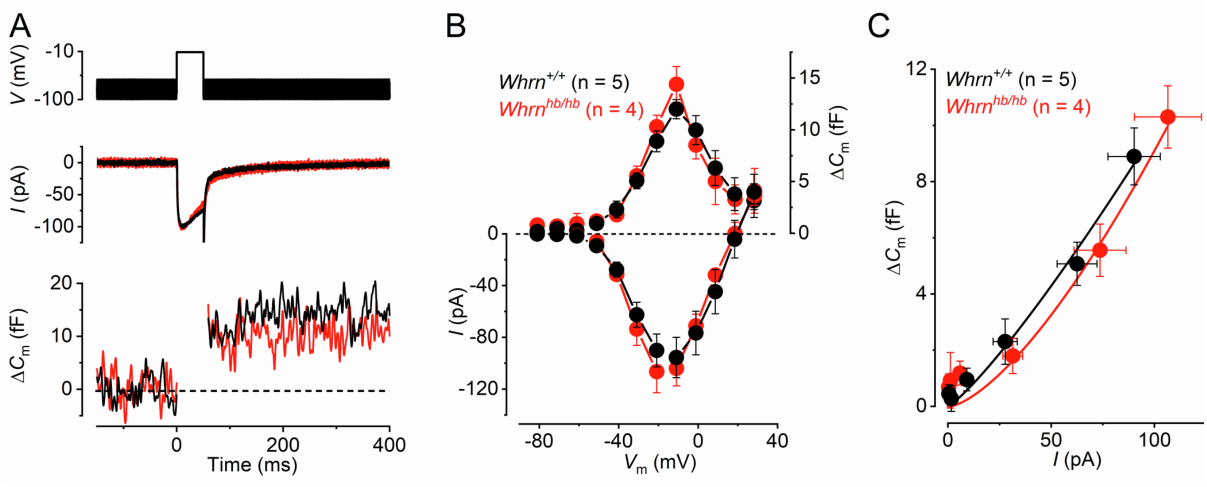

Figure S6. Ca^{2+} currents and ΔC_m in IHCs. I_{Ca} (A and B) and ΔC_m (A, bottom) responses in IHCs from *Whrn*$^{+/+}$ (n=5) and *Whrn*$^{hb/hb}$ (n=4) at P25, respectively. Recordings were obtained in response to 50 ms voltage steps, in 10 mV increments, from -81 mV. For clarity, only responses at -11 mV are shown in panel A. The voltage protocol is shown in the top panel above the traces. B-C Average peak I_{Ca} and ΔC_m curves from *Whrn*$^{+/+}$ and *Whrn*$^{hb/hb}$ IHCs. Synaptic transfer functions describing the relationship between the peak I_{Ca} and the corresponding ΔC_m from −71 mV to −21 mV. Fits to the data points are according to a power function: $\Delta C_m = const(I_{Ca}{}^{n})$. The power n was 1.19 ± 0.2 in *Whrn*$^{+/+}$ and 1.46 ± 0.4 in *Whrn*$^{hb/hb}$ (P = 0.1961). Data represent mean +/- SEM.

Table S6. Adjusted *P*-values for Tukey post-hoc multiple comparisons analysis using ANOVA of RQ values for each tissue against whirlin gene expression probe related to Figure 4.

Genotype	*ANOVA comparison*	*Gene expression probe*	*Tissue difference*	*Lower limit*	*Upper limit*	*P adjusted*
	Inner ears - Brain		0.503020	0.205030	0.801010	0.000250
	Eyes - Brain	*Whrn* exons 2-3	0.441730	0.143740	0.739720	0.001350
	Eyes - Inner ears		-0.061290	-0.359290	0.236700	0.975150
	Inner ears - Brain		0.612440	0.229240	0.995640	0.000520
	Eyes - Brain	*Whrn* exons 6-7	0.577010	0.193810	0.960210	0.001120
	Eyes - Inner ears		-0.035430	-0.418630	0.347770	0.998830
	Inner ears - Brain		0.541380	0.062470	1.020290	0.020320
**$Whrn^{+/+}$**	Eyes - Brain	*Whrn* exons 7-intron retention	0.178210	-0.300700	0.657120	0.818740
	Eyes - Inner ears		-0.363170	-0.842080	0.115740	0.209610
	Inner ears - Brain		0.417800	0.171460	0.664130	0.000230
	Eyes - Brain	*Whrn* exons 9-10	0.363190	0.116860	0.609520	0.001440
	Eyes - Inner ears		-0.054610	-0.300940	0.191730	0.967410
	Inner ears - Brain		0.674320	0.274200	1.074450	0.000250
	Eyes - Brain	*Whrn* exons 12-13	0.393580	-0.006550	0.793710	0.055680
	Eyes - Inner ears		-0.280740	-0.680870	0.119380	0.277400
	Inner ears - Brain		0.566940	0.372940	0.760950	0.000000
	Eyes - Brain	*Whrn* exons 2-3	0.276600	0.082600	0.470610	0.001820
	Eyes - Inner ears		-0.290340	-0.473250	-0.107430	0.000440
	Inner ears - Brain		0.196850	-0.088460	0.482150	0.300500
	Eyes - Brain	*Whrn* exons 6-7	-0.107290	-0.392600	0.178010	0.820420
	Eyes - Inner ears		-0.304140	-0.573130	-0.035150	0.019650
	Inner ears - Brain		2.748760	1.608700	3.888810	0.000000
**$Whrn^{hb/hb;BAC279+/-}$**	Eyes - Brain	*Whrn* exons 7-intron retention	-0.998150	-2.138200	0.141910	0.111270
	Eyes - Inner ears		-3.746900	-4.821760	-2.672050	0.000000
	Inner ears - Brain		-0.217180	-0.337220	-0.097130	0.000059
	Eyes - Brain	*Whrn* exons 9-10	-0.187840	-0.307880	-0.067790	0.000540
	Eyes - Inner ears		0.029340	-0.083840	0.142520	0.946290
	Inner ears - Brain		0.393980	0.106440	0.681520	0.002920
	Eyes - Brain	*Whrn* exons 12-13	-0.166930	-0.454470	0.120600	0.473120
	Eyes - Inner ears		-0.560910	-0.832000	-0.289820	0.000005
	Inner ears - Brain		0.847500	0.427390	1.267610	0.000017
	Eyes - Brain	*Whrn* exons 2-3	0.456070	0.035960	0.876180	0.027980
	Eyes - Inner ears		-0.391420	-0.811530	0.028690	0.077490
	Inner ears - Brain		0.801750	0.310400	1.293100	0.000410
	Eyes - Brain	*Whrn* exons 6-7	0.515050	0.023700	1.006400	0.036200
	Eyes - Inner ears		-0.286700	-0.778050	0.204650	0.456500
	Inner ears - Brain		3.825170	1.791540	5.858800	0.000052
**$Whrn^{hb/hb}$**	Eyes - Brain	*Whrn* exons 7-intron retention	-0.030910	-2.064540	2.002720	1.000000
	Eyes - Inner ears		-3.856070	-5.889700	-1.822440	0.000046
	Inner ears - Brain		0.277830	0.042940	0.512720	0.013970
	Eyes - Brain	*Whrn* exons 9-10	0.298730	0.063840	0.533620	0.007200
	Eyes - Inner ears		0.020900	-0.213990	0.255790	0.998980
	Inner ears - Brain		0.747540	0.298480	1.196610	0.000310
	Eyes - Brain	*Whrn* exons 12-13	0.313410	-0.135650	0.762480	0.281330
	Eyes - Inner ears		-0.434130	-0.883190	0.014930	0.062070
	Inner ears - Brain		0.567150	0.370260	0.764050	0.000000
	Eyes - Brain	Whrn exons 2-3	0.315080	0.118190	0.511970	0.000710
	Eyes - Inner ears		-0.252070	-0.448970	-0.055180	0.007430
	Inner ears - Brain		0.664640	0.465450	0.863830	0.000000
	Eyes - Brain	*Whrn* exons 6-7	0.393290	0.194100	0.592480	0.000044
	Eyes - Inner ears		-0.271350	-0.470540	-0.072160	0.004120
	Inner ears - Brain		3.161770	2.144810	4.178720	0.000000
**$Whrn^{hb/hb;BAC279-/-}$**	Eyes - Brain	*Whrn* exons 7-intron retention	-0.238740	-1.255690	0.778220	0.956930
	Eyes - Inner ears		-3.400500	-4.417460	-2.383550	0.000000
	Inner ears - Brain		0.136590	0.036930	0.236240	0.003870
	Eyes - Brain	*Whrn* exons 9-10	0.247640	0.147980	0.347290	0.000001
	Eyes - Inner ears		0.111050	0.011400	0.210700	0.023590
	Inner ears - Brain		0.616470	0.419800	0.813150	0.000000
	Eyes - Brain	*Whrn* exons 12-13	0.299730	0.093450	0.506000	0.002190
	Eyes - Inner ears		-0.316750	-0.523020	-0.110470	0.001200
	Inner ears - Brain		0.167070	-0.030350	0.364490	0.121060
	Eyes - Brain	*Whrn* exons 2-3	0.357910	0.171790	0.544040	0.000140
	Eyes - Inner ears		0.190840	-0.006570	0.388260	0.060950
	Inner ears - Brain		0.005100	-0.079830	0.090040	0.999730
	Eyes - Brain	*Whrn* exons 6-7	-0.020680	-0.100750	0.059400	0.932930
	Eyes - Inner ears		-0.025780	-0.110710	0.059160	0.886370
	Inner ears - Brain		0.114750	-0.001470	0.230980	0.053930
**$Whrn^{wi/wi}$**	Eyes - Brain	*Whrn* exons 7-intron retention	-0.123710	-0.233290	-0.014130	0.022650
	Eyes - Inner ears		-0.238460	-0.354690	-0.122240	0.000065
	Inner ears - Brain		-0.000020	-0.003210	0.003170	1.000000
	Eyes - Brain	*Whrn* exons 9-10	-0.000210	-0.003210	0.002800	0.999510
	Eyes - Inner ears		-0.000190	-0.003380	0.003000	0.999750
	Inner ears - Brain		0.225150	-0.029830	0.480130	0.098760
	Eyes - Brain	*Whrn* exons 12-13	0.393010	0.152610	0.633410	0.000880
	Eyes - Inner ears		0.167860	-0.087120	0.422850	0.309420

Table S7. Adjusted *P*-values for Tukey post-hoc multiple comparisons analysis using ANOVA of each genotype ∆CT means against whirlin gene expression probe and tissue related to Figure 4.

ANOVA comparison	*Gene expression probe*	*P adjusted*<br>*Brain*	 *Inner ears*	 *Eyes*
$Whrn^{+/+}$ vs $Whrn^{hb/hb;BAC279+/-}$		0.99999733	0.98298563	0.33689789
$Whrn^{+/+}$ vs $Whrn^{hb/hb}$		0.63236333	0.79449085	0.64602543
$Whrn^{+/+}$ vs $Whrn^{hb/hb;BAC279-/-}$		0.99687312	0.99975244	0.30611544
$Whrn^{+/+}$ vs $Whrn^{wi/wi}$		0.00000001	0.00000005	0.00000002
$Whrn^{hb/hb;BAC279+/-}$ vs $Whrn^{hb/hb}$	*Whrn* exons 2-3	0.62122019	0.96603013	0.99111583
$Whrn^{hb/hb;BAC279+/-}$ vs $Whrn^{hb/hb;BAC279-/-}$		0.99800275	0.99805576	0.99796809
$Whrn^{hb/hb;BAC279+/-}$ vs $Whrn^{wi/wi}$		0.00000000	0.00000001	0.00000053
$Whrn^{hb/hb}$ vs $Whrn^{hb/hb;BAC279-/-}$		0.87375664	0.90995583	0.95751506
$Whrn^{hb/hb}$ vs $Whrn^{wi/wi}$		0.00000022	0.00000000	0.00000041
$Whrn^{hb/hb;BAC279-/-}$ vs $Whrn^{wi/wi}$		0.00000007	0.00000010	0.00000756
$Whrn^{+/+}$ vs $Whrn^{hb/hb;BAC279+/-}$		0.08972870	0.99987609	0.34297787
$Whrn^{+/+}$ vs $Whrn^{hb/hb}$		0.25873359	0.99678748	0.23179412
$Whrn^{+/+}$ vs $Whrn^{hb/hb;BAC279-/-}$		0.47261001	0.99397650	0.07921702
$Whrn^{+/+}$ vs $Whrn^{wi/wi}$		0.00000000	0.00000000	0.00000000
$Whrn^{hb/hb;BAC279+/-}$ vs $Whrn^{hb/hb}$	*Whrn* exons 6-7	0.00032071	0.98524437	0.99633232
$Whrn^{hb/hb;BAC279+/-}$ vs $Whrn^{hb/hb;BAC279-/-}$		0.00203061	0.97936452	0.82100339
$Whrn^{hb/hb;BAC279+/-}$ vs $Whrn^{wi/wi}$		0.00000000	0.00000000	0.00000000
$Whrn^{hb/hb}$ vs $Whrn^{hb/hb;BAC279-/-}$		0.99901392	0.99998641	0.95314843
$Whrn^{hb/hb}$ vs $Whrn^{wi/wi}$		0.00000000	0.00000000	0.00000000
$Whrn^{hb/hb;BAC279-/-}$ vs $Whrn^{wi/wi}$		0.00000000	0.00000000	0.00000000
$Whrn^{+/+}$ vs $Whrn^{hb/hb;BAC279+/-}$		0.00000000	0.00000000	0.00000000
$Whrn^{+/+}$ vs $Whrn^{hb/hb}$		0.00000001	0.00000002	0.00000000
$Whrn^{+/+}$ vs $Whrn^{hb/hb;BAC279-/-}$		0.00000002	0.00000013	0.00000000
$Whrn^{+/+}$ vs $Whrn^{wi/wi}$		0.00002707	0.00083880	0.00000000
$Whrn^{hb/hb;BAC279+/-}$ vs $Whrn^{hb/hb}$	*Whrn* exon 7-intron retention	0.09567553	0.99888959	0.92498324
$Whrn^{hb/hb;BAC279+/-}$ vs $Whrn^{hb/hb;BAC279-/-}$		0.33067453	0.99686949	0.85560220
$Whrn^{hb/hb;BAC279+/-}$ vs $Whrn^{wi/wi}$		0.00000000	0.00000000	0.00000000
$Whrn^{hb/hb}$ vs $Whrn^{hb/hb;BAC279-/-}$		0.99132648	0.99997887	0.99924786
$Whrn^{hb/hb}$ vs $Whrn^{wi/wi}$		0.00000000	0.00000000	0.00000000
$Whrn^{hb/hb;BAC279-/-}$ vs $Whrn^{wi/wi}$		0.00000000	0.00000000	0.00000000
$Whrn^{+/+}$ vs $Whrn^{hb/hb;BAC279+/-}$		0.51237105	0.00562753	0.01139110
$Whrn^{+/+}$ vs $Whrn^{hb/hb}$		0.01864417	0.00070911	0.00158015
$Whrn^{+/+}$ vs $Whrn^{hb/hb;BAC279-/-}$		0.12283256	0.00013249	0.00310808
$Whrn^{+/+}$ vs $Whrn^{wi/wi}$		0.00000000	0.00000000	0.00000000
$Whrn^{hb/hb;BAC279+/-}$ vs $Whrn^{hb/hb}$	*Whrn* exons 9-10	0.00014748	0.84949950	0.86782315
$Whrn^{hb/hb;BAC279+/-}$ vs $Whrn^{hb/hb;BAC279-/-}$		0.00264807	0.33681937	0.87128307
$Whrn^{hb/hb;BAC279+/-}$ vs $Whrn^{wi/wi}$		0.00000000	0.00000000	0.00000000
$Whrn^{hb/hb}$ vs $Whrn^{hb/hb;BAC279-/-}$		0.97613232	0.88138389	0.99999502
$Whrn^{hb/hb}$ vs $Whrn^{wi/wi}$		0.00000000	0.00000000	0.00000000
$Whrn^{hb/hb;BAC279-/-}$ vs $Whrn^{wi/wi}$		0.00000000	0.00000000	0.00000000
$Whrn^{+/+}$ vs $Whrn^{hb/hb;BAC279+/-}$		0.35442398	0.99999976	0.06423007
$Whrn^{+/+}$ vs $Whrn^{hb/hb}$		0.00589785	0.70586466	0.00085195
$Whrn^{+/+}$ vs $Whrn^{hb/hb;BAC279-/-}$		0.05814346	0.81159284	0.01352044
$Whrn^{+/+}$ vs $Whrn^{wi/wi}$		0.00000052	0.00067895	0.00005191
$Whrn^{hb/hb;BAC279+/-}$ vs $Whrn^{hb/hb}$	*Whrn* exons 12-13	0.00001721	0.68339002	0.32044575
$Whrn^{hb/hb;BAC279+/-}$ vs $Whrn^{hb/hb;BAC279-/-}$		0.00047024	0.80093641	0.76789473
$Whrn^{hb/hb;BAC279+/-}$ vs $Whrn^{wi/wi}$		0.00000000	0.00046621	0.01991263
$Whrn^{hb/hb}$ vs $Whrn^{hb/hb;BAC279-/-}$		0.96553206	0.99997927	0.98805417
$Whrn^{hb/hb}$ vs $Whrn^{wi/wi}$		0.00574013	0.01119512	0.54267154
$Whrn^{hb/hb;BAC279-/-}$ vs $Whrn^{wi/wi}$		0.00217723	0.01440251	0.37495885

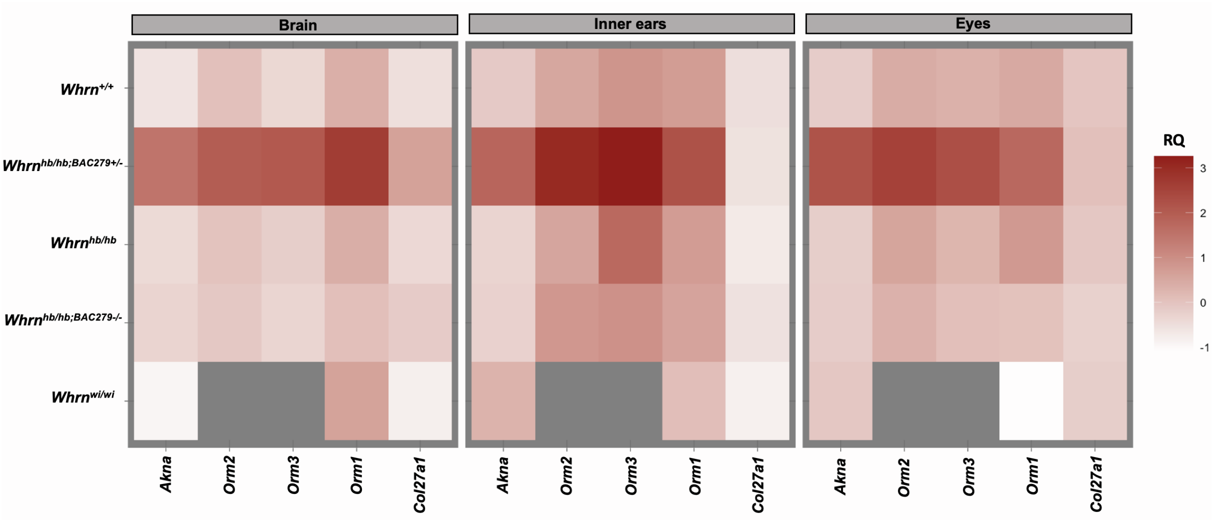

Figure S7. Expression profiles of *Akna*, *Orm2*, *Orm3*, *Orm1* and *Col27a1* related to Figure 4 and Table S8. Expression of additional genes in BAC279 were significantly increased ($P<0.05$), calculated from ΔCT means, between *Whrn*$^{hb/hb;BAC279+/-}$ (n=11) and each of the genotypes across all tissues with the exception of *Col27a1*, which was solely upregulated in brain. *Orm2* and *Orm3* expression are lacking from *Whrn*$^{wi/wi}$ (n=5) mutants as previously reported. Notably, no expression differences were identified between remaining genotypes (*Whrn*$^{+/+}$, n=8; *Whrn*$^{hb/hb}$, n=8; and *Whrn*$^{hb/hb;BAC279-/-}$, n=6) for these genes.

Table S8. Adjusted *P*-values for Tukey post-hoc multiple comparisons analysis using ANOVA of each genotype ∆CT means against the gene expression probe in BAC279 and tissue related to Figure S7.

ANOVA comparison	*Gene expression probe*	*Brain*	*P adjusted Inner ears*	*Eyes*
Whrn$^{+/+}$ vs *Whrn*$^{hb/hb;BAC279+/-}$		0.000000	0.000000	0.000000
Whrn$^{+/+}$ vs *Whrn*$^{hb/hb}$		0.795513	0.599794	0.997530
Whrn$^{+/+}$ vs *Whrn*$^{hb/hb;BAC279-/-}$		0.417044	0.872852	0.999946
Whrn$^{+/+}$ vs *Whrn*$^{wi/wi}$		0.393329	0.097757	0.606551
Whrn$^{hb/hb;BAC279+/-}$ vs *Whrn*$^{hb/hb}$	*Akna*	0.000000	0.000000	0.000000
Whrn$^{hb/hb;BAC279+/-}$ vs *Whrn*$^{hb/hb;BAC279-/-}$		0.000000	0.000000	0.000000
Whrn$^{hb/hb;BAC279+/-}$ vs *Whrn*$^{wi/wi}$		0.000000	0.000000	0.000000
Whrn$^{hb/hb}$ vs *Whrn*$^{hb/hb;BAC279-/-}$		0.950775	0.994863	0.999772
Whrn$^{hb/hb}$ vs *Whrn*$^{wi/wi}$		0.064323	0.005740	0.434133
Whrn$^{hb/hb;BAC279-/-}$ vs *Whrn*$^{wi/wi}$		0.020965	0.021502	0.591062
Whrn$^{+/+}$ vs *Whrn*$^{hb/hb;BAC279+/-}$		0.000000	0.000000	0.000000
Whrn$^{+/+}$ vs *Whrn*$^{hb/hb}$		0.945102	0.999251	0.886166
Whrn$^{+/+}$ vs *Whrn*$^{hb/hb;BAC279-/-}$		0.563654	0.664031	0.958279
Whrn$^{hb/hb;BAC279+/-}$ vs *Whrn*$^{hb/hb}$	*Orm2*	0.000000	0.000000	0.000000
Whrn$^{hb/hb;BAC279+/-}$ vs *Whrn*$^{hb/hb;BAC279-/-}$		0.000000	0.000000	0.000000
Whrn$^{hb/hb}$ vs *Whrn*$^{hb/hb;BAC279-/-}$		0.853898	0.733639	0.649057
Whrn$^{+/+}$ vs *Whrn*$^{hb/hb;BAC279+/-}$		0.000000	0.000006	0.000000
Whrn$^{+/+}$ vs *Whrn*$^{hb/hb}$		0.497404	0.157598	0.963335
Whrn$^{+/+}$ vs *Whrn*$^{hb/hb;BAC279-/-}$		0.977270	0.998669	0.375289
Whrn$^{hb/hb;BAC279+/-}$ vs *Whrn*$^{hb/hb}$	*Orm3*	0.000000	0.003480	0.000000
Whrn$^{hb/hb;BAC279+/-}$ vs *Whrn*$^{hb/hb;BAC279-/-}$		0.000000	0.000140	0.000000
Whrn$^{hb/hb}$ vs *Whrn*$^{hb/hb;BAC279-/-}$		0.770100	0.337299	0.614047
Whrn$^{+/+}$ vs *Whrn*$^{hb/hb;BAC279+/-}$		0.000000	0.000000	0.003625
Whrn$^{+/+}$ vs *Whrn*$^{hb/hb}$		0.999961	1.000000	0.904314
Whrn$^{+/+}$ vs *Whrn*$^{hb/hb;BAC279-/-}$		0.308191	0.980471	0.707430
Whrn$^{+/+}$ vs *Whrn*$^{wi/wi}$		0.573145	0.094740	0.001608
Whrn$^{hb/hb;BAC279+/-}$ vs *Whrn*$^{hb/hb}$	*Orm1*	0.000000	0.000000	0.037758
Whrn$^{hb/hb;BAC279+/-}$ vs *Whrn*$^{hb/hb;BAC279-/-}$		0.000000	0.000000	0.000213
Whrn$^{hb/hb;BAC279+/-}$ vs *Whrn*$^{wi/wi}$		0.000000	0.000000	0.000000
Whrn$^{hb/hb}$ vs *Whrn*$^{hb/hb;BAC279-/-}$		0.259967	0.979748	0.251142
Whrn$^{hb/hb}$ vs *Whrn*$^{wi/wi}$		0.634904	0.093784	0.000185
Whrn$^{hb/hb;BAC279-/-}$ vs *Whrn*$^{wi/wi}$		0.026771	0.288057	0.055441
Whrn$^{+/+}$ vs *Whrn*$^{hb/hb;BAC279+/-}$		0.000000	0.998321	0.653462
Whrn$^{+/+}$ vs *Whrn*$^{hb/hb}$		0.887149	0.751213	0.997348
Whrn$^{+/+}$ vs *Whrn*$^{hb/hb;BAC279-/-}$		0.129334	0.998300	0.126732
Whrn$^{+/+}$ vs *Whrn*$^{wi/wi}$		0.319553	0.528119	0.491216
Whrn$^{hb/hb;BAC279+/-}$ vs *Whrn*$^{hb/hb}$	*Col27a1*	0.000000	0.865197	0.441388
Whrn$^{hb/hb;BAC279+/-}$ vs *Whrn*$^{hb/hb;BAC279-/-}$		0.000021	0.999999	0.004870
Whrn$^{hb/hb;BAC279+/-}$ vs *Whrn*$^{wi/wi}$		0.000000	0.639111	0.051452
Whrn$^{hb/hb}$ vs *Whrn*$^{hb/hb;BAC279-/-}$		0.512080	0.923030	0.224152
Whrn$^{hb/hb}$ vs *Whrn*$^{wi/wi}$		0.069350	0.975872	0.669086
Whrn$^{hb/hb;BAC279-/-}$ vs *Whrn*$^{wi/wi}$		0.002984	0.727096	0.963983
